# Comparative Physio-Biochemical and Transcriptome Analyses Reveal Contrasting Responses to Magnesium Imbalances in Leaves of Mulberry (*Morus alba* L.) Plants

**DOI:** 10.3390/antiox13050516

**Published:** 2024-04-25

**Authors:** Yisu Shi, Xin Jin, Michael Ackah, Frank Kwarteng Amoako, Jianbin Li, Victor Edem Tsigbey, Haonan Li, Zipei Cui, Longwei Sun, Chengfeng Zhao, Weiguo Zhao

**Affiliations:** 1 Jiangsu Key Laboratory of Sericulture Biology and Biotechnology, School of Biotechnology, Jiangsu University of Science and Technology, Zhenjiang 212100, China; shiyisu1297@just.edu.cn (Y.S.); jinxin9502@126.com (X.J.); 13204812646@163.com (J.L.); tsigbeyv12@gmail.com (V.E.T.); czp0614@163.com (Z.C.); sunlongwei163@163.com (L.S.); inmitlessness@163.com (C.Z.); 2 Key Laboratory of Silkworm and Mulberry Genetic Improvement, Ministry of Agriculture and Rural Affairs, The Sericultural Research Institute, Chinese Academy of Agricultural Sciences, Zhenjiang 212100, China; 3 Institute of Plant Nutrition and Soil Science, Kiel University, Hermann-Rodewald-Straße 2, 24118 Kiel, Germany; kwamekwarteng242@gmail.com

**Keywords:** *Morus alba*, magnesium stress, antioxidants enzymes, chloroplast degradation, transcriptome, reactive oxygen species (ROS)

## Abstract

Magnesium (Mg) deficiency is a major factor limiting the growth and development of plants. Mulberry (*Morus alba* L.) is an important fruit tree crop that requires Mg for optimal growth and yield, especially in acid soils. However, the molecular mechanism of Mg stress tolerance in mulberry plants remains unknown. In this study, we used next-generation sequencing technology and biochemical analysis to profile the transcriptome and physiological changes of mulberry leaves under different Mg treatments (deficiency: 0 mM, low: 1 mM, moderate low: 2 mM, sufficiency: 3 mM, toxicity: 6 mM, higher toxicity: 9 mM) as T1, T2, T3, CK, T4, T5 treatments, respectively, for 20 days. The results showed that Mg imbalance altered the antioxidant enzymatic activities, such as catalase (CAT), peroxidase (POD), and superoxide dismutase (SOD), and non-enzymatic, including soluble protein, soluble sugar, malondialdehyde (MDA), and proline (PRO), contents of the plant. The Mg imbalances disrupted the ultrastructures of the vital components of chloroplast and mitochondria relative to the control. The transcriptome data reveal that 11,030 genes were differentially expressed (DEGs). Genes related to the photosynthetic processes (*CAB40*, *CAB7*, *CAB6A*, *CAB-151*, *CAP10A*) and chlorophyll degradation (*PAO*, *CHLASE1*, *SGR*) were altered. Antioxidant genes such as *PER42*, *PER21*, and *PER47* were downregulated, but *DFR* was upregulated. The carbohydrate metabolism pathway was significantly altered, while those involved in energy metabolism processes were perturbed under high Mg treatment compared with control. We also identified several candidate genes associated with magnesium homeostasis via RT-qPCR validation analysis, which provided valuable information for further functional characterization studies such as promoter activity assay or gene overexpression experiments using transient expression systems.

## 1. Introduction

Magnesium (Mg) is a plant growth macronutrient, and its deficiency has become a significant factor restricting intensive agricultural production, resulting in decreased crop yield and quality. Since Mg is also needed for human and animal diets, Mg nutrition in plants has become a significant concern for both food security and human health [1]. As a macronutrient, for optimal growth, plants require 1.5–3.5 g kg^−1^ dry matter of Mg, and this is known to be involved in many physiological and biochemical reactions [2]. Mg, after H, C, and O, is the fourth most prevalent element in plants; following N, P, K, and Ca, it is the eighth most plentiful mineral element on earth [3]. Both Mg deficiency and surplus should be considered when formulating management plans, as the level of Mg in the soil is essentially maintained by natural genesis and/or fertilization practices [4]. Soil with an abnormal Mg status, whether from Mg deficiency or excess, is thought to be detrimental to plant growth and development [5]. Mg plays a critical role in a broad range of enzymatic activities, including nucleotide metabolism and the cycle of nucleic acids in transcription, splicing, or replication, as part of its essential function in plant growth and development [4]. It is the core atom in the chlorophyll molecules of photosynthesizing organisms and a co-factor necessary to activate more than 300 enzymes [6,7]. Even though Mg is crucial for plant development and growth, it receives less attention than other macronutrients such as N, P, and K, consequently giving it the name “the forgotten element” [8,9]. 

Mg deficiency (MGD) or oversupply in plants is a widespread problem confronting the quality and productivity of the agricultural system [9]. Research has examined and exposed the impacts of Mg deficiency on plant physiology, such as biomass partitioning, CO_2_ assimilation, photooxidative defense, and net CO_2_ assimilation [10]. Intriguingly, Mg deficiency stimulates nutrient translocation and the expression of genes involved in the defense response in immature leaves [10]. Interveinal chlorosis is a symptomatic indicator of insufficient Mg due to chlorophyll breakdown. Even though Mg is a relatively mobile atom, remobilization occurs from older to younger mature leaves [11]; hence, young mature leaves are typically more susceptible to Mg insufficiency, as seen by their rapid starch accumulation, Mg concentration reduction, and chlorophyll decline [12]. Usually, interveinal chlorosis occurrences are species-specific; for instance, in *Arabidopsis*, it took more than 2 weeks to be seen [12]. A reduction in phloem sucrose export and sugar buildup in the source leaves is an additional significant early indicator of Mg deficiency. Responses to Mg deficiency, such as increased sucrose buildup, interrupted photosynthetic activity, and even observable morphological abnormalities, as triggered by Mg deficiency, require different times in various plant species [13].

Both Mg deficiency and excess stress conditions impact plant morphogenesis, development, and yield via distinct pathways [14]. In addition, Mg-induced stress conditions have a significant role in activating the plant stress response, which includes phytohormones, downstream metabolism (C-fixation), ion absorption, and antioxidant activities [15]. Mg deficiency or excessive supply also generate reactive oxygen species (ROS) and cell damage due to excess photosynthetic electrons, and so it is imperative to investigate various levels of Mg-induced stress and the gene expression response at the whole genomic level to help understand Mg deficiency or excess supply response mechanisms in mulberry and make critical decisions to maximize production and avoid yield loses.

Mulberry is an important economic crop plant which plays a key role in the sericulture industry [16]. Despite its usage in sericulture as main source of food for the silkworm (*Bombyx mori*), it remains an integral part of Chinese medicine and ecological and environmental objectives and is widely cultivated worldwide in subtropical and temperate regions [17]. To date, global transcriptomic analysis related to Mg imbalances in mulberry are nonexistent. To the best of our knowledge, this is the first global transcriptomic study analysis of mulberry in response to Mg imbalances. An early study on Mg stress in mulberry has been reported in the past [18], revealing oxidative and antioxidants responses in mulberry response to Mg imbalance. However, the earlier-reported investigation lacks genetic evidence and key gene pathways mechanisms in mulberry’s response to Mg stresses, and, hence, gene expression and the mechanism involved in Mg stress in mulberry is not clear. Studies reveal that, at the transcript level, the gene encoding *chlorophyll a/b binding protein 2* (*CAB2*) was downregulated and linked to Mg deficiency [10]. In this study, we used genome-wide RNA-seq technology to investigate the transcript expressions level in mulberry’s response to Mg imbalances. RNA-seq is an effective method for identifying genes and clarifying gene function connected with Mg availability by studying phenotypic alterations. In our previous study [19], we thoroughly investigated Mg supply (deficiency, low, and toxicity) on the plant morpho-physiological and metabolomic response in mulberry plants. We hypothesized that the disruption caused by Mg imbalance would alter higher gene regulation involved in metabolic alterations in chlorophyll biosynthesis and defense mechanisms in *M. alba* plants. The aim of this current investigation was first to examine the impact of Mg deficiency (MGD), low, excess, and sufficiency on the physio-biochemical (enzymes and non-enzymes) status of mulberry plants with respect to their growth and development, and, secondly, to elucidate the molecular mechanism involving Mg supply and deficiency in mulberry plants’ response to Mg imbalances. The current research would play a pivotal role in comprehending transcript expression and enzymatic and biochemical activities in response to Mg nutrient imbalances in mulberry. Furthermore, it will provide a theoretical foundation for conducting genomic studies on mulberry, which could be of utmost importance to mulberry genetic breeding programs.

## 2. Materials and Methods

### 2.1. Mulberry Plant (Morus alba), Materials, Growth Conditions, and Magnesium Treatment

Mulberry (Yu-711), a member of the *M. alba* L (white mulberry) species, was obtained from the National Mulberry GenBank at Jiangsu University of Science and Technology in Zhenjiang, Jiangsu, China. The mulberry plants’ growth and Mg treatments (applied ones) were carried out in a controlled environment in accordance with the methods described in our previous study [19]. Mulberry plants were administered with different Mg levels (deficiency: 0 mM, low: 1 mM, moderate low: 2 mM, sufficiency: 3 mM, toxicity: 6 mM, higher toxicity: 9 mM) as T1, T2, T3, CK, T4, T5 treatments, respectively. A sample of 3–5 leaves in all groups were sampled at 20 days (d) of the experiment (showing Mg symptoms) and temporarily stored in a −80 °C freezer for further analysis.

### 2.2. Physiological and Biochemical Determination in M. alba Response to Mg Imbalances

The mulberry leaves in all groups were collected to evaluate the contents of non-enzymatic parameters including proline (PRO), soluble protein, soluble sugar, malondialdehyde (MDA), and chlorophyll (Chl). Furthermore, the enzymatic activity of peroxidase (POD), superoxide dismutase (SOD), and catalase (CAT) was also examined. These parameters were determined using a detection kit supplied by Suzhou Keming Biotechnology Co., Ltd., Suzhou, China. All mulberry samples’ preparation and testing procedures adhered to the company’s recommendations, and each indicator utilized three biological replicates. 

### 2.3. Chloroplast and Mitochondria Ultra-Microstructure by TEM Analysis

To analyze the chloroplast and mitochondria ultra-microstructure of *M. alba* leaves’ response to Mg imbalances, Mg-treated *M. alba* leaves samples in each concentration mentioned above were prepared. Fresh *M. alba* leaves were fixed overnight in a 2.5% glutaraldehyde phosphate buffer. The leaves were then rinsed with 0.1M PBS twice for 15 min each time. Afterwards, 1% H_2_[OsO_4_(OH_2_)] was added for 1h for immobilization. The leaves were then rinsed with 0.1 M PBS twice again for 15 min each time. Dyeing was performed by staining the leaves with 2% uranium acetate solution for 30 min. This was then followed by dehydration with 50%, 70%, and 90% anhydrous ethanol for 15 min each time. This was followed by a one-time dehydration for 20 min. Tissue infiltration was performed by mixing a 1:1 volume ratio of anhydrous acetone and embedding agent and shaking for 2 h. After the pure embedding agent penetrated the tissue and was shaken for 2 h, the tissue with the pure embedding agent was polymerized in the oven for 2 h at 37 °C, 24 h at 45 °C, and 48 h at 60 °C. The tissues were then trimmed, and ultra-thin slices (approx. 120 nm) were prepared and stained with 4% uranium acetate for 20 min and lead citrate for 5 min. Finally, the stained ultra-thin sections were placed on single-hole copper mesh and observed under TECNAI 10 transmission electron microscope and photographed.

### 2.4. RNA Extraction, Library Construction and Sequencing

Mulberry leaves from the six groups of Mg supply and deficiency treatment, deficiency (T1), low (T2, T3), sufficiency (CK), and excess (T4, T5) [19], were used for the RNA extraction, complementary DNA library construction, and sequencing. Total RNA was extracted using a Trizol reagent kit (Invitrogen, Carlsbad, CA, USA) according to the manufacturer’s protocol. RNA quality was assessed on an Agilent 2100 Bioanalyzer (Agilent Technologies, Palo Alto, CA, USA) and checked using RNase free agarose gel electrophoresis. After total RNA was extracted, eukaryotic mRNA was enriched by Oligo(dT) beads (rRNA was removed by Ribo-Zero^TM^ Magnetic Kit (Epicentre, Madison, WI, USA)). Then, the enriched mRNA was fragmented into short fragments using a fragmentation buffer and reversely transcribed into cDNA using an NEBNext Ultra RNA Library Prep Kit for Illumina (NEB #7530, New England Biolabs, Ipswich, MA, USA). The purified double-stranded cDNA fragments were end-repaired, had a base added, and were ligated to Illumina sequencing adapters. The ligation reaction was purified with the AMPure XP Beads (1.0X) (Beckman Coulter Life Sciences, Shanghai, China). Ligated fragments were subjected to size selection by agarose gel electrophoresis and were polymerase chain reaction (PCR) amplified. The resulting cDNA library was sequenced using Illumina Novaseq6000 from Gene Denovo Biotechnology Co., Guangzhou, China. 

### 2.5. RNA-Seq Data Analysis and Bioinformatics

After RNA sequencing, the raw reads were filtered to obtained clean reads using fastp v0.18.0 [20] to remove reads containing adapters and reads containing more than 10% of unknow nucleotides (N) and to remove low quality reads containing more than 50% of low quality (Q-value ≤ 20) bases. The short reads alignment tool bowtie2 v2.2.8 [21] was used in mapping reads to the ribosome RNA (rRNA) database. The rRNA mapped reads were removed and the remaining clean reads were further used in assembly and gene abundance calculation. Furthermore, the obtained paired-end clean reads were mapped to the *Morus notabilis* reference genome using HISAT2. 2.4 [22] with “-rna-strandness RF” and other parameters set as a default. Quantification of gene abundance was achieved by assembling mapped reads of each sample using StringTie v1.3.1 [23,24] in a reference-based approach. For each transcription region, a FPKM (fragment per kilobase of transcript per million mapped reads) value was calculated to quantify its expression abundance and variations, using RSEM [25] software v1.3.3. Correlation and principal component (PCA) analysis of the samples and the replicas were performed by R software v4.2.

Analysis of differentially expressed genes (DEGs) was performed by the DESeq2 [26] software v1.42.1. Gene expressions which met the criteria for the false discovery rate (FDR < 0.05) and log2 fold change, |log2FC|>=1, were considered differentially expressed genes/transcripts. Heatmap and volcano plots were performed on the DEGs using R packages. Gene Ontology (GO) (http://geneontology.org/ accessed on 15 December 2023) enrichment analysis was conducted, and the calculated p-value went through FDR correction, taking FDR ≤ 0.05 as a threshold. GO terms meeting this condition were defined as significantly enriched GO terms in DEGs. KEGG pathways enrichment (Kyoto Encyclopedia of Genes and Genomes) was also conducted using the same criteria as the GO analysis.

### 2.6. RT-qPCR Gene Validation and Analysis

To verify the DEGs identified by high-throughput sequencing, 30 DEGs were selected for real-time quantitative polymerase chain reaction (RT-qPCR) validation. We followed the protocol described in our previous study for the RT-qPCR analysis [17]. Mulberry leaves samples used for the RNA-seq analysis were also used for total RNA and cDNA synthesis for qRT-PCR validation. In addition, fold changes in gene expression were estimated using the 2^−ΔΔCt^ method [27]. The primers and the gene names used for the RT-qPCRs validation are listed in Table S1.

### 2.7. Subcellular Localization of Difunctional Dihydroflavonol 4-Reductase/Flavanone 4-Reductase (DFR) Gene

To investigate the site of expression of the *DFR* gene, the plant-mPLoc website was used to predict the localization of the gene. After the prediction, the *DFR* gene was PCR amplified using the primers in Table S1, and the correct sequence of DFR showing the correct band at the desired location was sent to Wuhan Boyuan Biotechnology Co., LTD, Wuhan, China, for the subcellular localization analysis. To investigate the subcellular localization of the gene, an *Arabidopsis thaliana* protoplast transformation experiment was utilized. Several samples of unbolted Arabidopsis seedlings were taken, and 10 mL of enzymolysis solution was added to immerse the tissue completely and allowed to stand at 24 °C for 4 h. Afterwards, filtration was performed with a 40 μm filter, the content centrifuged at 300 rpm for 3 min, and the supernatant discarded. The content was then washed with 10 mL of pre-cooled W5 solution twice, and centrifuged at 300 rpm, 4 °C for 3 min. The protoplast suspension was prepared by taking 100 μL protoplast and 500 μL MMG solution. A mixture of 100 μL protoplast suspension, 20 μL recombinant plasmid, and 120 μL PEG4000 solution was made and gently mixed and left for 30 min at room temperature. The reaction was then terminated by diluting it with 1 mL of W5. The protoplasts were collected by centrifugation at 300 rpm for 3 min, and the supernatant was discarded. This was followed by washing with 1 mL W5 twice, and, finally, 1 mL of W5 solution was added and cultured at 28 °C for 24 h in a dark environment. After culturing, the supernatant was removed, leaving about 100 μL protoplasts for laser confocal microscope observation.

### 2.8. Statistical Analyses of Physiological and Biochemical

All statistical analyses were performed with R software v4.2. A significant difference test was performed with ANOVA using Turkeys at *p* ≤ 0.05 in all cases. RT-qPCR, physiological and biochemical analysis figures were visualized via tools in Hiplot Pro (https://hiplot.com.cn/ accessed on 10 February 2024), a comprehensive web service for biomedical data analysis and visualization.

## 3. Results

### 3.1. Physiological and Biochemical Response to Mg Supply and Deficiency in Mulberry Plants

**Figure 1:**
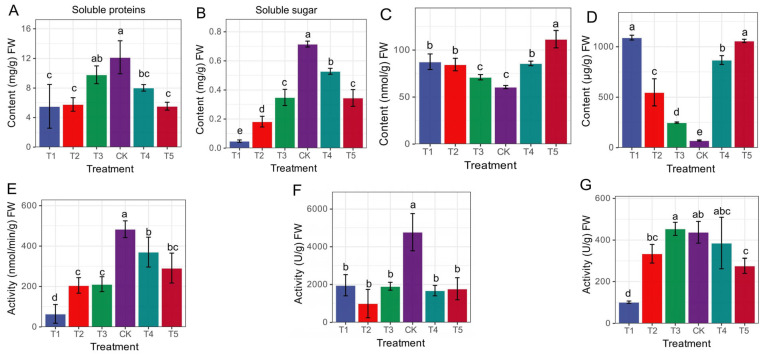
Physiological and biochemical responses to Mg supply (low and high) and deficiency in *M. alba* after 20 days of treatment. (**A**) Soluble protein; (**B**) soluble sugar; (**C**) malondialdehyde (MDA); (**D**) proline (PRO); (**E**) catalase (CAT); (**F**) peroxidase (POD); (**G**) superoxide dismutase (SOD). Different letters above the bar represent significant differences (*p* < 0.05) using Tukey test. T1: 0 mM Mg, T2: 1 mM Mg, T3: 2 mM Mg, CK: 3 mM Mg, T4: 6 mM Mg, T5: 9 mM Mg.

The morpho-physiological data for this study have already been dealt with and published in our previous study [19], to which this work is a continuation. To supplement transcriptome analysis, non-enzymatic indicators such as PRO content, soluble protein content, soluble sugar content, and MDA content were assessed. Additionally, our analysis of enzyme activities, namely POD, SOD, and CAT, demonstrated that the imposition of Mg stress on mulberry plants led to a range of biochemical modifications. Compared to the CK group, all the treatment groups (T1–T5) recorded a decrease in soluble protein and soluble sugar content, with the most affected occurring in the T1 and T5 groups. The CK group was significantly higher than the deficiency and toxicity groups (Figure 1A,B). We observed that soluble sugar content significantly decreased in the Mg-deficiency and low-Mg-supply groups (T1, T2) compared to the Mg-toxicity (T4, T5) groups (Figure 1B). Again, within the MDA and PRO content, Mg-deficiency and low supply groups (T1–T3) and the toxicity (T4 and T5) groups significantly recorded the highest content in both MDA and PRO (Figure 1C,D) compared to the CK, which significantly decreased in content. The results suggest that Mg deficiency, low supply and toxicity significantly decreased the content of soluble sugars in Mg toxicity, PRO, and MDA above the sufficient-Mg-fed mulberry plants. Surprisingly, the CK groups’ treatment increased enzyme CAT (Figure 1E), POD (Figure 1F), and SOD (Figure 1G) activities. Compared to the control, SOD and POD activities significantly decreased in the Mg-deficiency (T1), low supply (T2, T3), and toxicity (T4, T5) groups. Mg toxicity significantly increased CAT activity compared to the deficiency and low supply groups. Interestingly, the POD activity in the Mg toxicity, deficiency and low supply groups were significantly not different. The activity of SOD behaved differently from POD and CAT. We observed that SOD activity responded to the low Mg supply and toxicity level by significantly increasing activity (Figure 1G). It was evident that the SOD activity decreased significantly in the Mg-deficiency group (T1) but increased in the Mg supply groups, whether low (T2, T3), optimal (CK) or toxic level (T4, T5). 

### 3.2. Effects of Magnesium Stress on Chloroplast Ultra-Microstructure of Mulberry Leaves

**Figure 2:**
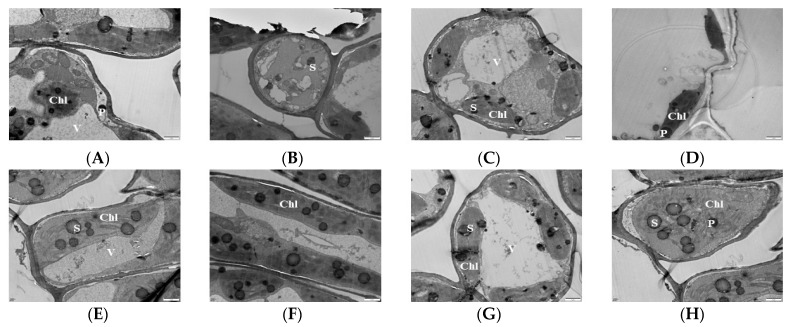
Effects of magnesium (MgSO_4_) stress on chloroplast ultra-microstructure of mulberry leaves. (**A**,**B**) 0 mM; (**C**) 1 mM; (**D**) 2 mM; (**E**,**F**) 3 mM; (**G**) 6 mM; (**H**) 9 mM. Chl: chloroplast; S: starch grains; P: starvation granules; V: vacuole.

Chloroplasts are the sites of photosynthesis in plants, and the complete structure of chloroplasts is a prerequisite for normal photosynthesis in plants. The more basal and basal lamellae in the chloroplasts of plant leaves, the neater and denser the arrangement of the lamellae and the stronger the photosynthesis ability of the plant leaves. Transmission electron microscopy (TEM) was used to observe the chloroplasts of mulberry leaf samples treated with Mg stress for 20 d. The results showed that, at the 0 mM (T1), the chloroplasts were deformed and irregular in shape; some were spherical and had damaged films. The basal granules were disintegrated and there were no basal lamellae, indicating that MGD led to a significant reduction in the stacking degree of leaf thylakoid films and the enlargement of chloroplast starch grains and starvation granules (Figure 2A,B). At 1 mM (T2), the basal lamellae of the chloroplasts partially disintegrated, with some of the chloroplasts being spherical. Some of the chloroplasts were broken and separated, and the basal grains became loose, but the double membrane was clearly visible (Figure 2C). With the supplementation of 2 mM Mg, the basal lamellae of the chloroplast began to disintegrate and remained fusiform and became loose compared to that of the control (Figure 2D). In the 3 mM Mg treated leaves, the chloroplast became a regular spindle close to the cell wall and surrounded the inner surface of the cell. The bilayer membrane was clearly visible. Also, the basal stroma sheets were neat and regular, and the thylakoids were numerous and tightly stacked (Figure 2E,F). The shape of the chloroplast (6 mM Mg-treated leaves) remained unchanged, the basal stroma was only slightly damaged, and it was all continuously surrounded by cells. Also, the basal stroma layer did not change much compared with the control; only the starch grains became larger (Figure 2G). The structure of the chloroplast at 9 mM was damaged, the basal lamellae were loose, and the starch grains became larger and tended to disintegrate (Figure 2H).

### 3.3. Effects of Magnesium Stress on Mitochondrial Ultra-Microstructure of Mulberry Leaves

**Figure 3:**
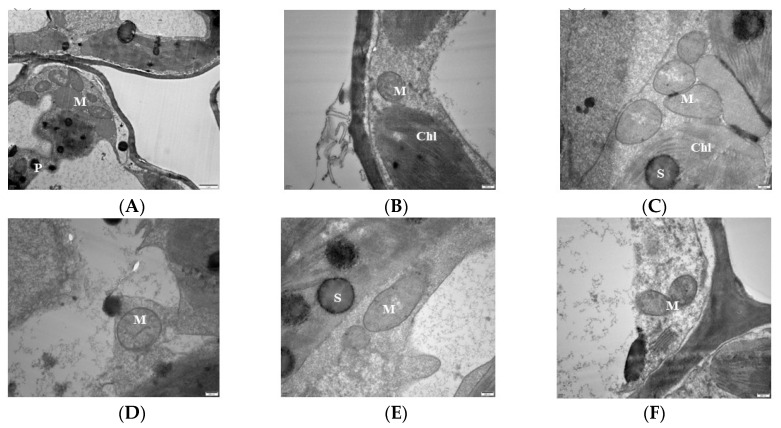
Effects of magnesium (MgSO_4_) stress on mitochondrial ultra-microstructure of mulberry leaves. (**A**) 0 mM C; (**B**) 1 mM; (**C**) 2 mM; (**D**) 3 mM; (**E**) 6 mM; (**F**) 9 mM. M: mitochondria; Chl: chloroplasts; S: starch grains; P: starvation granules.

Analysis of the mitochondrial ultra-microstructure shows that most of the mitochondrial inner lumen at 0 mM treatment expanded, the structure lost integrity, the membrane system disappeared, there was no clear boundary with the surrounding matrix, and the number of cristae was reduced compared with CK (Figure 3A). The mitochondria of 1 mM were deformed, hollow, and membrane-blurred, and the cristae was unevenly distributed (Figure 3B). The 2 mM mitochondria begin to disintegrate but remained short rods or spherical, and the distribution of the cristae was still visible but become loose compared to the control (Figure 3C). In the 3 mM-treated leaves, the mitochondrial structure of the leaf cells was intact with a uniform matrix, the envelope was clear, and the crest was uniformly distributed and structurally intact (Figure 3D). The mitochondrial bilayer membrane was damaged, and the mitochondria were partially deformed, but the overall structure did not change much, and the distribution of the crest could still be seen in the 6 mM treatment (Figure 3E). The 9 mM-treated leaf mitochondria were deformed, hollow, and membrane-blurred, and the number of cristae decreased. The cristae distribution was uneven, but the overall structure remained intact (Figure 3F).

### 3.4. Transcriptome Profiling and Quality Control in M. alba Response to Mg Imbalances

**Figure 4:**
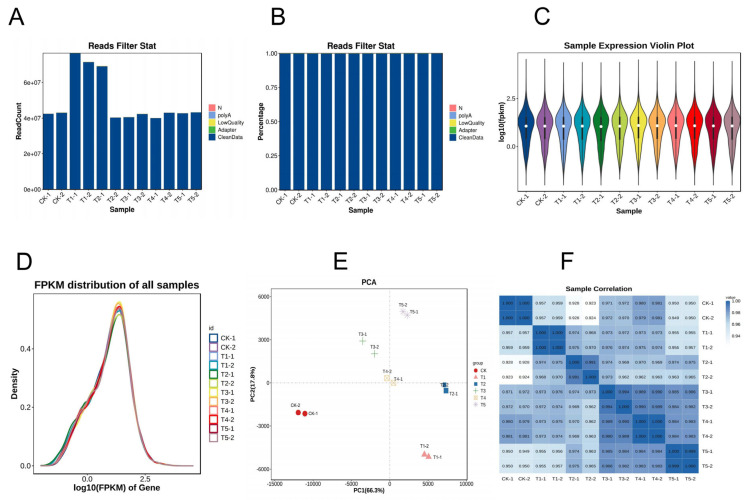
Sequence statistics of the transcriptome data: (**A**) reads count filtering; (**B**) clean reads filtering (%); (**C**) sample violin expression plot; (**D**) density plot of FPKM; (**E**) principal component (PC) analysis; (**F**) sample correlation heatmap analysis. Mg sufficiency (CK; 3 mM), deficiency (T1; 0 mM), low (T2; 1 mM), moderate low (T3; 2 mM), toxicity (T4; 6 mM), and high toxicity (T5; 9 mM).

To investigate how Mg imbalances affect gene regulation in *M. alba*, a transcriptome profile study was carried out on the mulberry leaves under deficient low, toxicity, and supply of Mg for 20 d of the experiment and were used for RNA sequencing and analysis. A total of 595,041,834 raw reads were obtained from the 24 libraries, with an average of 49,586,820 reads per sample (Table S2; Figure 4A) through Illumina sequencing. After filtering the raw sequencing data, a total of 593,776,014 of clean data, averaging 49,481,334.5 and constituting 99.79% of the clean reads, were obtained from the samples (Figure 4B). After base composition analysis of the clean reads, the GC content of the sample reads (on average) was 45.94%, and the Q20 and Q30 scores were 97.64% and 93.41%, respectively (Table S2). In addition, more than 70% of the clean reads on average (Table S2) were uniquely mapped to the mulberry *M. notabilis* reference genome using Hisat2 software v2.4 [22]. The alignment of the sample’s clean reads to the reference genome mostly occurred in the exonic region and then intergenic and intron (Figure S2). The gene expression distribution of the samples is shown in Figure 4C,D. Principal component analysis (PCA) was conducted using the normalized counts to visualize the similarity of the data sets/profiles. PCA results revealed that samples of the same group clustered together, and PC1 showed a 66.3% variation occurred between the sample groups (Figure 4E). A Pearson’s correlation heatmap of the samples indicated that the correlation coefficient (R^2^) was more than 0.9, indicating the reliability of the data samples (Figure 4F).

### 3.5. Analysis of Differentially Expressed Genes (DEGs) in Mulberry Response to Mg Supply

**Figure 5:**
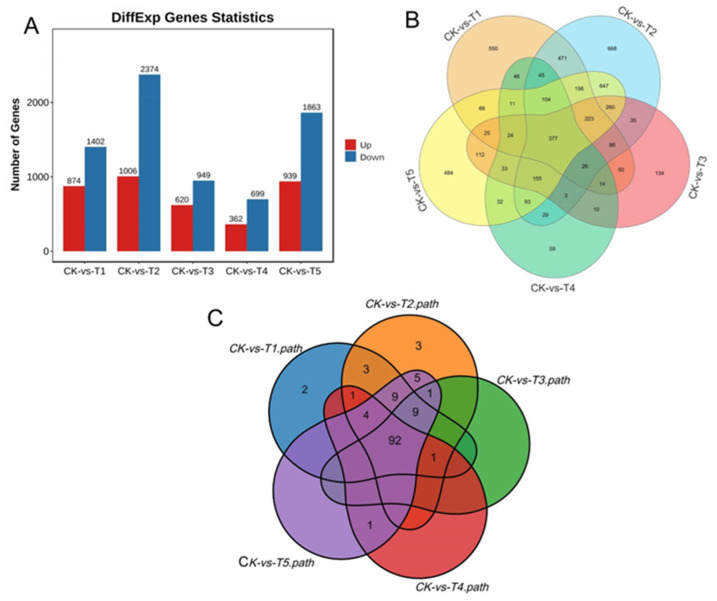
Overview of RNA-seq data. (**A**) Different gene expression statistics; (**B**) Venn diagram depicting the relationship of the DEGs among the five treatments groups; (**C**) Venn diagram indicating the relationship KEGG pathways of the DEGs among the five treatments groups.

Under the various Mg treatments and deficiencies, we observed 28,504 unigenes, and 11,088 were DEGs in all the treatments comparison with the sufficiency (CK) (Figure 5A). We identified 2276 (20.53%) DEGs in the Mg-deficiency treatment (CK-vs-T1), 3380 (30.48%) DEGs in the low Mg supply (CK-vs-T2), and 1569 (14.15%) DEGs in CK-vs-T3 (Figure 5A). In the Mg toxicity levels, we identified 1061 (9.57%) and 2802 (25.27%) DEGs in CK-vs-T4 and CK-vs-T5, respectively (Figure 5A). We further analyzed the DEGs from each treatment in comparison with the sufficiency group (CK) to visualize the DEGs situation in the various treatments (CK-vs-T1, CK-vs-T2, CK-vs-T3, CK-vs-T4, and CK-vs-T5), using a Venn diagram analysis to observe the DEGs within and between the treatments (Figure 5B). We observed that 550 of the DEGs were specifically expressed in Mg deficiency (CK-vs-T1), 668 in low Mg supply (CK-vs-T2), 134 in CK-vs-T3, 59 in Mg toxicity (CK-vs-T4), and 484 in higher Mg toxicity (CK-vs-T5) (Figure 5B; Table 1). We observed again that 377 DEGs were commonly expressed in all treatments. Furthermore, 112 DEGs were observed between Mg toxicity and Mg deficiency, and 521 DEGs between Mg deficiency and low Mg supply (Figure 5B). Volcano plots and cluster heatmap analysis reveal the pattern of the DEGs in each treatment group in comparison with the control. In the Mg-deficiency treatment, 847 DEGs increased in status and 1402 DEGs decreased in status, and genes in the same expression status clustered together (Figure S2A,B). Within the low-Mg-supply group (T2), 1006 DEGs were upregulated, whereas 2374 were downregulated (Figure S2C,D). A similar trend occurred in the moderately low supply group (T3), where 620 DEGs were up- and 949 were downregulated (Figure S2E,F). In the Mg toxicity group, 362 DEGs were upregulated, and 699 DEGs were downregulated in T4 (Figure S2G,H). In CK-vs-T5, 939 and 1863 DEGs were upregulated and downregulated, respectively (Figure S2I,J), suggesting that Mg imbalances altered more downregulation of genes in mulberry leaves.

### 3.6. Analysis of Chlorophyll Synthesis- and Photosynthesis-Related DEGs

**Figure 6:**
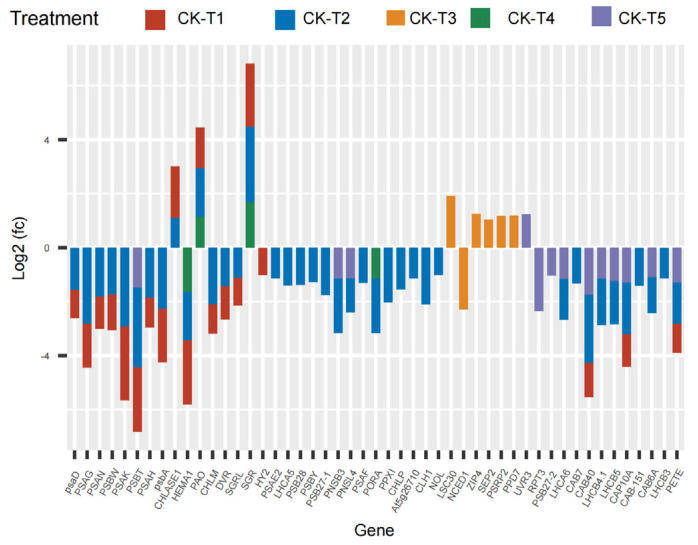
Chlorophyll synthesis- and photosynthesis-related DEGs response to Mg imbalances in mulberry leaves at 20 days of Mg treatment. DEGs in Mg sufficiency (CK) in comparison with the other treatments are as follows. CK-vs-T1: DEGs in Mg-deficient plants; CK-vs-T2, CK-vs-T3: DEGs in low-Mg-treated plants; CK-vs-T4, CK-vs-T5: DEGs in Mg-toxicity plants.

The current results reveal that several photosynthetic and chlorophyll synthesis genes were altered in mulberry’s response to Mg imbalances. From the results screened, 16 DEGs were found in CK-vs-T1, 29 DEGs in CK-vs-T2, seven DEGs in CK-vs-T3, five DEGs in CK-vs-T4, and six DEGs in CK-vs-T5 (Figure 6), suggesting that Mg-deficiency and low-Mg-treated plants induced more photosynthetic and chlorophyll genes DEGs compared to the toxicity groups. Several genes related to photosystem I and II reaction centers and core complex (*PSAG*, *PSAD*, *PSAK*, *PSAH*, *PSAN*, *PSBW*, *psba*), among the DEGs were significantly downregulated and expressed only in Mg-deficiency and low-Mg-treated (CK-vs-T2) plants, whereas *PSBT* was downregulated, and only in Mg-deficiency, CK-vs-T2, and CK-vs-T5 leaves. More phytochromobilin, ferredoxin oxidoreductase (*HY2*), was altered and downregulated only in the Mg-deficiency group. Chlorophyll-synthesis related genes including Light-Harvesting complex 5 (*LHCA5*) associated with photosystem I, protochlorophyllide oxidoreductase (*PORA*), Mg-protoporphyrin IX methyltransferase (*CHLM*), glutamyl-tRNA reductase 2 (*HEMA1*), and many others were downregulated specifically in CK-vs-T1 and CK-vs-T2 leaves. Interestingly, *LHCA5*, photosystem I and II reaction center and core complex genes (*PSAF*, *PSAE2*, *PSB28*, *PSBY*), and protoporphyrinogen oxidase 1 (*PPXI*), *CLH1* (chlorophyllase-1), Chlorophyll(ide) b reductase (*NOL*), etc., were altered only in CK-vs-T2 and were downregulated. Surprisingly, chlorophyll synthesis- and photosynthesis-related genes in CK-vs-T3 had no expression in relation to other treatments. All the genes, including Ferritin-3 (*LSC30*), stress enhanced protein 2 (*SEP2*), and 30S ribosomal protein 2 (*PSRP2*), in CK-vs-T3 were upregulated except *NCED1* (9-cis-epoxycarotenoid dioxygenase), which was downregulated. Moreover, chlorophyll degradation genes, such as pheophorbide a oxygenase (*PAO*), chlorophyllase-1 *(CHLASE1)*, protein STAY-GREEN *(SGR)*, were upregulated in response to Mg deficiency, low (CK-vs-T2) and toxicity (CK-vs-T4) levels. Chlorophyll a-b binding proteins genes (*CAB40*, *CAB7*, *CAB6A*, *CAB-151*, *CAP10A*, etc.) were expressed and downregulated in CK-vs-T1, CK-vs-T2 and the toxicity group (CK-vs-T5). Surprisingly, three genes including DNA photolyase (*UVR3*), root phototropism protein 3 (*RPT3*), and photosystem II D1 precursor processing protein (*PSB27-2*) were all altered only in the higher Mg toxicity leaves (CK-vs-T5). Nevertheless, photosynthetic NDH subunit of subcomplex *B 3* (*PNSB3*) and photosynthetic NDH subunit of lumenal location 4 (*PNSL4*) were altered and downregulated in response to Mg toxicity (CK-vs-T5) and low-Mg-treated leaves (CK-vs-T2, Figure 6).

### 3.7. Analysis of Cytochrome DEGs in Morus alba Responses Mg Imbalances

**Figure 7:**
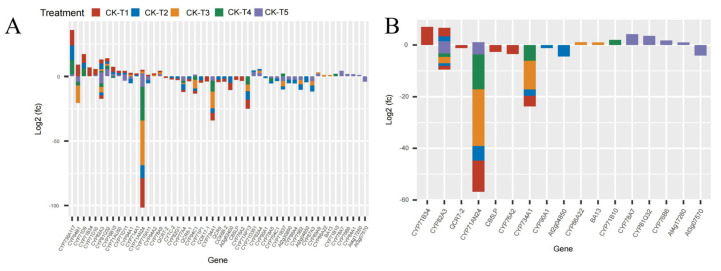
The cytochrome DEGs response to Mg imbalances in mulberry leaves at 20 days of Mg treatment. (**A**,**B**) cytochrome DEGs. DEGs in Mg sufficiency (CK) in comparison with the other treatments are as follows. CK-vs-T1: DEGs in Mg-deficient plants; CK-vs-T2, CK-vs-T3: DEGs in low-Mg-treated plants; CK-vs-T4, CK-vs-T5: DEGs in Mg-toxicity plants.

Mg plays a critical role as a cofactor in many enzymes, including cytochrome P450 enzymes (CYP450) [28] and cytochrome protein synthesis [29]. In this study, several CYP450 genes were observed across the treatment groups. In the MGD-treated plants (CK-vs-T1), 41 DEGs (17 up- and 24 downregulated) were expressed, 45 DEGs were expressed (11 up and 34 down) in CK-vs-T2, 26 DEGs (10 up and 16 down) in CK-vs-T3, 20 (six up and 14 down) DEGs in CK-vs-T4, and 47 DEGs (21 up and 26 down) in CK-vs-T5 (Figure 7). Among these, the CYP71 family (*CYP71D9*, *CYP71AU50*, *CYP71AN24*, *CYP71AP13*, *CYP71B34*, *CYP71D10*, *CYP71D11*, *CYP71B10*, *CYP71P1*, *CYP71B37*, *CYP71D351*) was the most predominant. Also, the CYP94 family (*CYP94A1*, *CYP94A2*, *CYP94B1*, *CYP94C1*), CYP82 family (*CYP82A3*, *CYP82A4*, *CYP82G1*), CYP76 family (*CYP76A2*, *CYP76B10*, *CYP76B6*), CYP86 (*CYP86A4*, *CYP86A22*), and other single families (Figure 7A,B) were observed. Most of the CYP71 family exhibited up- and downregulation patterns in CK-vs-T1, CK-vs-T2, and CK-vs-T5. Several mulberry accessions *CYP71AN24* isoforms were observed and downregulated. 

### 3.8. Analysis of Cell Wall- and Carbohydrates-Related DEGs

**Figure 8:**
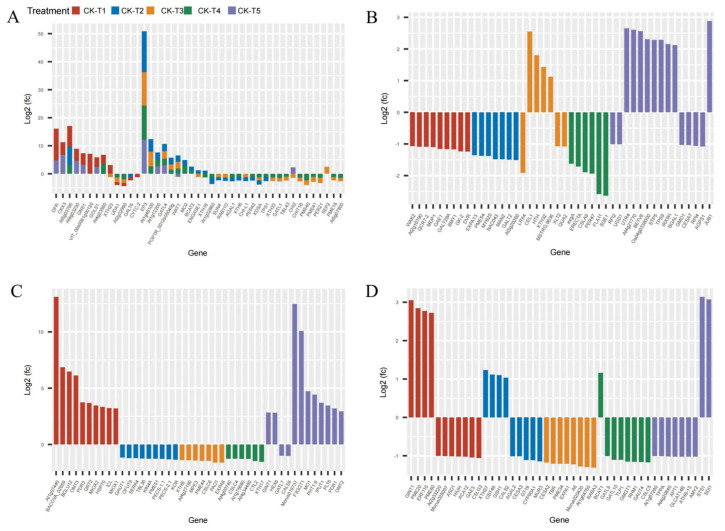
Cell wall- and carbohydrates-related DEGs response to Mg imbalances in mulberry leaves at 20 days of Mg treatment (**A**–**D**).

Provision of Mg and MGD treatments altered several DEGs related to carbohydrates and the cell wall. From the transcriptome analysis, 185 DEGs were recorded from MGD, 155 DEGs in CK-vs-T2, 71 DEGs in CK-vs-T3, 55, and 370 DEGs were obtained from CK-vs-T4 and CK-vs-T5, respectively. The results indicate that a large portion of the DEGs were related to carbohydrates and cell wall transcripts. We therefore screened and analyzed the top 50 expression genes including down- and upregulated genes from each treatment group (Figure 8). Comparison of the DEGs in the CK-vs-T1 with the toxicity groups reveal that six genes, including *CKX3* (cytokinin dehydrogenase 3; XP_010090055.1), *DFR* (bifunctional dihydroflavonol 4-reductase/flavanone 4-reductase; XP_010110052.1), *GNS1* (glucan endo-1,3-beta-glucosidase, basic isoform; XP_010090235.1), *GOLS1* (galactinol synthase 1; XP_010100446.1), *At4g03230* (G-type lectin S-receptor-like serine/threonine-protein kinase; EXB47196.1), and *At4g23560* (hypothetical protein L484_006703; EXC34348.1) were expressed only in these groups (Figure 8A–D). Further, Mg-treated plants expressed some genes which were absent in the MGD plants. These genes include *EP3* (endochitinase EP3; XP_010094102.1), *GATL4* (probable galacturonosyltransferase-like 4; XP_010105689.1), and *WAT1* (plant-drug/metabolite exporter; MSTRG.6737) and were all upregulated. Genes (*XTH33*, *XTH16*, *XTH6*) encoding probable xyloglucan endotransglucosylase/hydrolase proteins, and *PER42*, *PER21*, *PER47* encoding peroxidase, were predominantly altered in low-Mg-treated (CK-vs-T3) and toxicity groups (CK-vs-T4). Again, genes such as *PME44*, *PME40*, *PME41*, *PME18*, *PME51*, *PME43*, *PME54*, etc., encoding pectinesterase/pectinesterase inhibitor proteins were altered in Mg-treated leaves and were downregulated. However, *PME20* and *PME60* were only expressed in MGD-treated leaves and were significantly upregulated (Figure 8A–D).

### 3.9. Analysis of Ubiquitin-Related DEGs in Morus alba Responses Mg Imbalances

**Figure 9:**
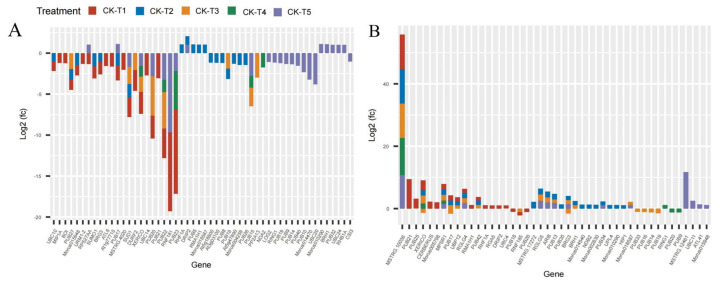
Analysis of ubiquitin-related DEGs response to Mg imbalances in mulberry leaves at 20 days of Mg treatment (**A**,**B**).

Several E3 ubiquitin-protein ligase and U-box domain-containing protein genes were differentially expressed in mulberry leaves in response to Mg levels. From our analysis, 41 DEGs (25 down-, 16 upregulated) were identified in CK-vs-T1. In the low-Mg-treated leaves, CK-vs-T2 observed 40 DEGs (15 down-, 25 upregulated), whereas CK-vs-T3 produced 29 DEGs (16 down-, 13 upregulated) (Figure 9A,B). Further, the toxicity-treated leaves observed 11 DEGs (seven down-, four upregulated) in CK-vs-T4, whereas in CK-vs-T5, 38 DEGs (21 up- and 17 downregulated) were observed. Curiously, comparing the DEGs between MGD leaves (CK-vs-T1) and toxicity leaves (CK-vs-T5) treatments, two genes including *RPS27AA* (ubiquitin; XP_010107560.1) were downregulated in CK-vs-T1 but upregulated in CK-vs-T5, whereas *RNF181*(E3 ubiquitin-protein ligase RING1-like protein) was exclusively altered in MGD and higher Mg toxicity (CK-vs-T5) leaves. However, *PUB23* (E3 ubiquitin-protein ligase; EXC12998.1) was uniquely altered in the MGD and toxicity groups (Figure 9A,B). Furthermore, *BOI* (E3 ubiquitin-protein ligase BOI isoform X1), *URM1-2* (Ubiquitin-related modifier 1-2-like protein), *ATL6* (E3 ubiquitin-protein ligase; XP_010094662.1), and *MSTRG.4020* (probable E3 ubiquitin-protein ligase XBOS32), *UBC14* (Ubiquitin-conjugating enzyme E2 14) were altered only in the MGD leaves and downregulated. Meanwhile, *PUB25* (U-box domain-containing protein 25), *CERBERUS* (Putative E3 ubiquitin-protein ligase LIN-1), *Morus005798* (U-box domain-containing protein 1; XP_010103043.1), *RGA5* (U-box domain-containing protein 4) were exclusively altered and upregulated in the MGD leaves. Nevertheless, gene *PUB21* encoded by *Morus019341* (U-box domain-containing protein 21; XP_010091753.1) and *Morus019305* (XP_010105562.1 U-box domain-containing protein 21) in mulberry, with the former showing upregulation and the latter exhibiting downregulation, were remarkably altered in the MGD leaves. On the other hand, *RING1*(E3 ubiquitin-protein ligase Praja-2; XP_010100889.1) and *PUB9* (U-box domain-containing protein 9; XP_010109324.1) were altered in toxicity groups but not in the low Mg or MGD groups. Exclusively, *CSI3* (U-box domain-containing protein 13; EXB60107.1) and *LOG2* (E3 ubiquitin-protein ligase MGRN1; EXC16229.1) were altered and downregulated in the higher toxicity treatment (CK-vs-T5), whereas *UBC11* (Ubiquitin-conjugating enzyme E2 11; EXC16959.1), MSTRG.1246*1* (E3 ubiquitin-protein ligase RNF25 isoform X2), *ATL41* (E3 ubiquitin-protein ligase; XP_010087307.1), *PUB32* (U-box domain-containing protein 32; EXC19149.1), *UBC24* (probable ubiquitin-conjugating enzyme E2 24; XP_010108186.1), and *RHB1A* (probable E3 ubiquitin-protein ligase; XP_010112939.1) were upregulated in the same treatment (CK-vs-T5). Some of the genes were expressed in all the treatment comparisons. For instance, *MSTRG.10036* (E3 ubiquitin-protein ligase RNF25 isoform X2) and *MPSR1* (E3 ubiquitin-protein ligase; XP_010108676.1) were significantly overexpressed in all the treatments (Figure 9A,B).

### 3.10. Analysis of Transcriptional Factors (TFs) DEG Families

**Figure 10:**
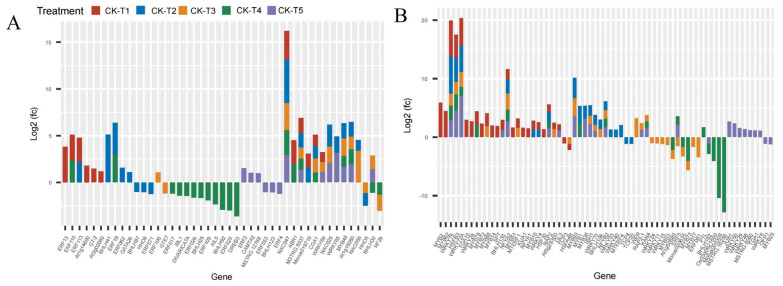
Analysis of DEGs of transcriptional factor families in *M. alba* response to Mg imbalances (**A**,**B**).

The analysis of the transcriptome data uncovered that the supplementation of Mg and the MGD groups altered the expression of several families of TFs. Remarkably, 92, 105, 67, 40, and 80 DEGs belonging to TFs were observed in CK-vs-T1, CK-vs-T2, CK-vs-T3, CK-vs-T4, and CK-vs-T5, respectively. Due to the large size of the DEGs of TFs, we screened the first 40 DEGs based on the highest expression level from each treatment group (Figure 10). From the analysis, the notable TFs families included 16 *ERF* (ethylene-responsive transcription factor), 16 *MYB*, 10 *BHLH*, 16 *WRKY*, 3 *NAC,* and 3 *HSF* (heat stress transcription factor) and were observed to be more prominently expressed in this investigation (Figure 10A,B). Interestingly, all the TFs (including *ERF13*, *ERF113*, *ERF110*, *GT-2*, *NAC047*, *WRKY69*, *WRKY72A*, *MYB2*, *MYB62*, *BHLH130,* and many others) in the MGD-treated levels were upregulated, whereas *TCP3* and *HSFA4B* were downregulated. In the Mg toxicity groups, five TF genes, including *ERF053*, *BHLH123*, *ERF4*, *MYB25* and *OFP1*, were downregulated and only expressed in CK-vs-T5. However, most of the TFs in CK-vs-T3 and CK-vs-T4 were downregulated compared to the other treatments. *BHLH62* was only altered and downregulated in the toxicity groups, whereas *WRKY75*, *HSFB3*, *WRKY72A*, *NFYA2*, *NAC047,* and *MSTRG.5037* were overexpressed in all the treatments (Figure 10A,B). 

### 3.11. Analysis of Signaling and Plant Hormones Related DEGs in Morus alba Responses to Mg Imbalances

**Figure 11:**
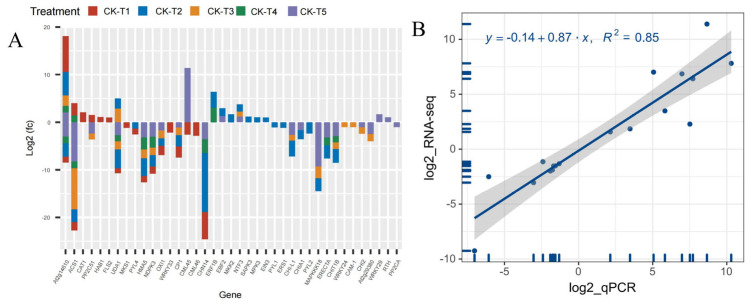
(**A**) Analysis of DEGs related to signaling and plant hormones. (**B**) Regression line plot showing quantitative real-time polymerase chain reaction (RT-qPCR) for the relative expression level and RNA sequencing (RNA-seq) results of the differentially expressed genes (DEGs). The mulberry *actin3* (HQ163775.1) gene was used as the internal control gene. All the samples were conducted with three independent biological replicates and three technical replicates. The relative expression levels were calculated as 2^−∆∆CT^, where ∆∆CT = ΔCT (a target sample) − ΔCT (a reference sample). The *y* axis represents the log2FC of RNA-Seq, and the *x* axis denotes the log2 fold change in relative expression obtained by RT-qPCR.

Analysis of the DEGs related to signaling and plant hormones reveal that 39 DEGs were altered across the treatment (MGD, low and toxicity). In the MGD group, 19 DEGs (six up- and 13 downregulated) were observed. In the low (CK-vs-T2), 26 DEGs including 10 up- and 16 downregulated were screened, whereas 17 DEGs (14 down-, three upregulated) were recorded in CK-vs-T3. However, in the toxicity groups, there were 11 DEGs including eight down- and three upregulated in CK-vs-T4, whereas CK-vs-T5 observed 22 DEGs comprising 16 downregulated, five upregulated and one exhibiting both up- and downregulation patterns (Figure 11A). Genes including *CAT1* (*Catalase isozyme 1*; *EXC51646.1*), *HAB1* (protein phosphatase 2C 77; XP_010090094.1), and *FLS2* (LRR receptor-like serine/threonine-protein kinase isoform X1; XP_010097599.1) were upregulated, whereas *MKS1* (*protein MKS1*; *XP_010090872.1*), *WRKY33* (putative WRKY transcription factor 33; EXB54274.1), and *CML46* (putative calcium-binding protein CML45; EXB31951.1) were downregulated, and all these genes were exclusively altered in MGD treated leaves (Figure 11A). Meanwhile, we observed that *Morus008596* encoding *CML45* (probable calcium-binding protein CML45; XP_010089908.1) was overexpressed in the higher Mg toxicity group; however, *Morus015667* encoding *CML45* (putative calcium-binding protein CML30; *EXC19992.1*) was downregulated. Remarkably, *WRKY29* (WRKY transcription factor 22; XP_010112624.1), *RTH* (protein RTE1-HOMOLOG; XP_010113481.1), and *PP2CA* (protein phosphatase 2C 37; XP_010096621.1) were only altered and upregulated in CK-s-T5, except *PP2CA,* which was downregulated. Further analysis reveals that two mulberry genes, *Morus012919* encoding *ACS1* (1-aminocyclopropane-1-carboxylate synthase 1; XP_010092578.1) and *Morus024218* encoding *ACS1* (1-aminocyclopropane-1-carboxylate synthase; XP_010089045.1), were significantly altered in all the treatments. The former was upregulated in MGD and CK-vs-T4 leaves but downregulated in CK-vs-T3 and CK-vs-T5, whereas the latter was downregulated in all the treatments. Other genes, including *NDPK3* (*nucleoside diphosphate kinase 3*; *XP_010093431.1*), *CP1* (calmodulin; *XP_010109984.1*), and *OXI1* (serine/threonine-protein kinase; XP_010100801.1), were downregulated in either the MGD, low or toxic groups (Figure 11A). The expression patterns of these genes in each group in comparison with the CK are shown in Figure 11A.

### 3.12. RNA Sequencing Validation by RT-qPCR

To validate the RNA-seq analysis, 20 DEGs were randomly selected, and then RT-qPCR analysis was performed (Table S1). As shown in Figure 11B, a strong positive correlation coefficient (r^2^ = 0.85) between the expression levels obtained from the RNA-Seq data and RT-qPCR was observed (T1–T5), which clearly proved the reliability of the RNA-seq data.

### 3.13. GO Category Enrichment Analysis of the DEGs in Mulberry Plants under Mg Supply and Mg Deficiency

To investigate the functional significance of the DEGs involved in Mg imbalances, we performed a GO enrichment analysis test with *p* and *FDR* values ≤ 0.05. Based on these criteria, the top 20 GO terms and enrichments involving the DEGs were analyzed and classified. The GO terms were classified into biological process (BP), cellular component (CC), and molecular function (MF) (Figures S3 and S4). In the MGD-treated leaves, the top 20 GO terms based on significant (FDR ≤ 0.05) reveal that BP and MF were crucial to MGD treatment. GO terms such as response to chitin (GO:0010200), response to drug (GO:0042493), and mitochondrial ATP synthesis coupled electron transport (GO:0042775) were significantly implicated (Figure S3A). Also, in the MF, several GO terms (16) including dioxygenase activity (GO:0051213), hydrolase activity, hydrolyzing O-glycosyl compounds (GO:0004553), oxidoreductase activity, acting on paired donors, with incorporation or reduction of molecular oxygen (GO:0016705), structural constituent of ribosome (GO:0003735), heme binding (GO:0020037), and many others were significant in MGD (Figure S3A, Table S3). The top 20 enrichment analysis involving the DEGs in the MGD leaves reveals that DEGs such as *PUB20*, *PUB22*, *PUB23*, *ATL6* (encoding E3 ubiquitin-protein ligase and U-box domain-containing proteins), *WRKY53*, *WRKY11*, *WRKY53*, *ERF109*, *HSF30* (transcription factors), etc., were significantly enriched in response to chitin GO term in the BP. Further, *NDB2* (putative NADH-ubiquinone oxidoreductase; EXB30583.1), *QCR7-2* (cytochrome b-c1 complex subunit 7; XP_010110339.1), *QCR*9 (cytochrome b-c1 complex subunit 9; XP_010097831.1), *QCR6-1* (Cytochrome b-c1 complex subunit 6; EXB97277.1), *CYTC-2* (cytochrome c; XP_010104528.1), etc., were significantly enriched in the mitochondrial ATP synthesis coupled electron transport in the BP, suggesting their involvement in energy and defense mechanisms (Figure S3B; Table S3). Several GO terms were enriched with the DEGs in the MF. For instance, heme binding was enriched with 57 DEGs, 263 DEGs were enriched in oxidoreductase activity, and 59 DEGs were enriched in structural constituent of ribosome (Figure S3B; Table S3) These genes, including *CAT1*, *CYP82A3*, *CYP79A68*, *CYP76B10*, *PER54*, *PER66*, *CYP710A11*, *CYP94B1*, *CYP76A2*, *CYP71AN24*, *CYP71AU50*, *DOX2*, *RPL34*, *IAA13*, *PER21,* and many others, were enriched in several MF GO terms (Table S3).

In the Mg-treated plants, the DEGs were enriched in all the three GO categories (BP, MF, and CC). In low Mg supply, polysaccharide metabolic process (GO:0005976), cell wall organization, or biogenesis (GO:0071554) were significant in the BP in CK-vs-T2 of the top 20 GO terms. However, response to chitin, response to biotic stimulus (GO:0009607), and response to other organism (GO:0051707) were significant to BP in CK-vsT3. Cell wall (GO:0005618), cell periphery (GO:0071944), extracellular region (GO:0005576), intrinsic component of plasma membrane (GO:0031226), and many others were significant to CC in CK-vs-T2 and CK-vs-T3; however, photosystem (GO:0009521), photosystem I (GO:0009522) and plant-type cell wall (GO:0009505) were peculiar to CK-vs-T2 (Figure S3C,E). In the MF, protein kinase activity (GO:0004672), hydrolase activity, hydrolyzing O-glycosyl compounds, hydrolase activity, acting on glycosyl bonds (GO:0016798), UDP-glycosyltransferase activity (GO:0008194), catalytic activity (GO:0003824), etc., were significant in either CK-vs-T2 or CK_vs-T3 (Figure S3C,E). Enrichment analysis of the GO terms involving the DEGs is shown in Figure S3D,F. From the enrichment analysis, several DEGs were enriched in the GO terms. For instance, 157 DEGs (*CHI-L1*, *PME41*, *PME22*, *WAT1*, *XTH23,* etc.) were enriched in polysaccharide metabolic process, 162 DEGs (*GATL1*, *EP3*, *CSLD2* and many more) in cell wall organization or biogenesis, 515 DEGs in cell wall, 36 DEGs in photosystem, and 1636 DEGs in catalytic activity (Figure S3D,F; Tables S4 and S5). In the Mg toxicity groups, similar GO terms and enrichment trends were seen as observed in the low-Mg-supply groups (Tables S6 and S7). However, more DEGs were enriched in these groups (Figure S4A–D). Analysis of the secondary GO terms level reveal that 25 subgroups’ (cellular process, metabolic process, response to stimulus, signaling, growth, detoxification, etc.) GO terms were under BP, 22 subgroups’ (cell, cell part, membrane, membrane part, extracellular region, etc.) GO terms were in the CC, and 14 subgroups’ (binding, catalytic activity, signal transduction activity, structural molecule activity, antioxidant, nutrient reservoir activity, etc.) GO terms were in the MF (Figure S4E). 

### 3.14. KEGG Enrichment Analysis of the DEGs in Mulberry Plants under Mg Deficiency and Mg Supply

To further understand the pathways mechanisms of the DEGs, we performed KEGG enrichment using the KEGG main public database to unravel the significant metabolic or transduction pathways enriched with the DEGs using the same criteria q ≤ 0.05. The analysis reveals that in the MGD comparison with CK (CK-vs-T1), 21.92% (499/2276) of DEGs were mapped to 121 KEGG pathways. Furthermore, in the low Mg supply, the analysis reveals that in CK-vs-T2, 20.38% (689/3380) of DEGs were annotated to 128 KEGG pathways. Again, 18.04% (283/1569) of DEGs were mapped to 103 pathways in CK-vs-T3. In the Mg toxicity treatments, we observed that, in CK-vs-T4, 19.23% (204/1061) of DEGs were linked to 99 pathways. In addition, 19.09% (535/2802) were mapped to 121 KEGG pathways in CK-vs-T5 (Table S8). Furthermore, the pathways of the DEGs were compared among the treatment groups (CK-vs-T1, CK-vs-T2, CK-vs-T3, CK-vs-T4, and CK-vs-T5). We observed that 92 pathways were common to all the treatment groups, two pathways (sulfur relay system and proteasome) were only linked to CK-vs-T1, and three pathways (C5-branched dibasic acid metabolism, one carbon pool by folate, and ribosome biogenesis in eukaryotes) were only linked to CK-vs-T2 (Figure 5C, Table S8). 

Based on significant q-value ≤ 0.05 and biological significance, there were 41 KEGG pathways that were significantly enriched across the treatments (Figure S5). Among them, the ribosome pathway (ko03010) was significantly enriched with 46 DEGs in the MGD (CK-vs-T1) (Figure S5A,B). In the low-Mg-supply groups, photosynthesis—antenna proteins with 11 DEGs (ko00196), amino sugar and nucleotide sugar metabolism with 38 DEGs (ko00520), metabolic pathways with 368 DEGs (ko01100), flavonoid biosynthesis with 21 DEGs (ko00941), biosynthesis of secondary metabolites (223 DEGs) (ko01110), glycosphingolipid biosynthesis—globo and isoglobo series (seven DEGs) (ko00603), sesquiterpenoid and triterpenoid biosynthesis (13 DEGs) (ko00909), glycosaminoglycan degradation (eight DEGs) (ko00531), galactose metabolism (15 DEGs) (ko00052), and photosynthesis (17 DEGs) (ko00195) were significantly enriched in CK-vs-T2 (Figure S5C,D; Table S8). Further, in CK-vs-T3, biosynthesis of secondary metabolites (110 DEGs), MAPK signaling pathway—plant (18 DEGs) (ko04016), starch and sucrose metabolism (18 DEGs) (ko00500), isoflavonoid biosynthesis (four DEGs) (ko00943), cyanoamino acid metabolism (11 DEGs) (ko00460), diterpenoid biosynthesis (nine DEGs) (ko00904), etc., were significantly enriched (Figure S5G,H; Table S8). In the Mg toxicity groups, almost similar pathways were observed in CK-vs-T4 (Figure S5J,K) and CK-vs-T5 (Figure S5M,N). Notably, plant hormone signal transduction, phenylpropanoid biosynthesis, photosynthesis—antenna proteins, MAPK signaling pathway—plant, diterpenoid biosynthesis, etc., were significantly enriched (Table S8). Analysis of the secondary classification of the pathways reveals that the metabolism pathway is sub-grouped into 11 subgroups (global overview, carbohydrates, lipid metabolism, energy metabolism, etc.), genetic information processing into four subgroups (folding, sorting and degradation, replication and repair, translation, transcription), environmental information processing into two subgroups (signal transduction, membrane transport), cellular processes into one (transport and catabolism), and organismal systems into one (environmental adaptation) (Figure S5C–O) in all the treatments. 

### 3.15. Subcellular Localization of MaDFR Protein in Mulberry

**Figure 12:**
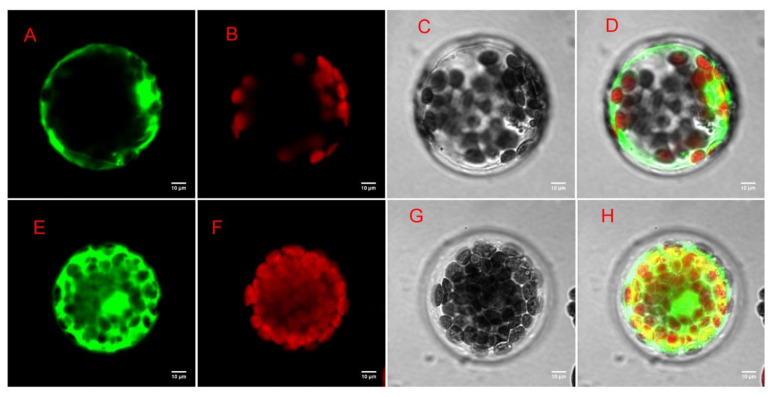
Subcellular localization of *MaDFR* gene. (**A**) Target protein fluorescent channel; (**B**) chloroplast fluorescence channel; (**C**) brightfield; (**D**) overlay image; (**E**) empty vector control fluorescence channel; (**F**) chloroplast fluorescence channel; (**G**) brightfield; (**H**) overlay image.

To further investigate the function of the DEGs genes in *Morus alba*, difunctional dihydroflavonol 4-reductase/flavanone 4-reductase (*DFR*) gene, which was involved in many GO functions, including catalytic activity, UDP-glucose 4-epimerase activity, oxidoreductase activity, carbohydrate metabolic processes, and KEGG pathways including flavonoid biosynthesis, biosynthesis of secondary metabolites, etc., was chosen as candidate gene for further exploration via subcellular localization analysis. Based on the presence of a targeted sequence in the protein, the predictive result from the plant-mPLoc website showed that the *MaDFR* protein was predicted to be localized in the cytoplasm. The result of the subcellular localization prediction was evaluated experimentally, using the *Arabidopsis thaliana* protoplast transformation method and the reporter green fluorescent protein (GFP). The recombinant plasmid pan580-egfp-MaDFR was transformed into *Arabidopsis thaliana* seedlings. The results showed that the experimental data are identical with the plant-mPLoc website predicted ones. Using laser confocal microscopy, *Arabidopsis* cells transformed with the recombinant plasmids; however, the green fluorescence of egfp-MmDFR was observed in the cytoplasm, and the expression effect was good (Figure 12).

## 4. Discussion

Abiotic factors including drought, heat, light, hypoxia, nutrition, etc., affect the transcriptional (mRNAs) level and translational (proteins) level of plants and hence affect their growth and development. Mg nutrient, an indispensable macronutrient, and the supply of Mg at the right proportion is crucial to plant growth. This is because Mg aids in metal ions transport and enzyme activation, which is essential for plant growth and development. The light-capturing complex of chloroplasts relies on Mg as a fundamental component of chlorophyll pigments, which is essential for the assimilation of CO_2_ during photosynthesis [30]. The present study investigated the physiological and molecular mechanisms of Mg supply and deficiency in *M. alba.* To achieve this objective, we conducted a physiological study and transcriptome analysis on mulberry seedlings that were exposed to distinct levels of Mg supply (sufficiency, low, and toxicity) as well as Mg deficiency (zero Mg) for a period of 20 days. 

### 4.1. Mg Imbalances Affect Mulberry Plant Growth by Changing Chlorophyll and Photosynthesis Related Genes Content

Chlorophyll molecules comprising Chl *a* and Chl *b* form the component of photosynthesis complex in plants. Chl plays a key role in the capture, transmission, and transformation of light energy [31] that the plant can use for its growth and metabolism and therefore forms essential part of growth and development. In our previous study [19], we revealed captivating evidence that mulberry response to MGD, low, or toxicity levels (stresses) remarkably reduced Chl (Chl *a*, Chl *b* and total Chl) contents in mulberry leaves after 20 d of treatment. This situation resulted in the decline in overall photosynthetic machinery (including net photosynthesis, transpiration rate, etc.) and in the plant’s biomass in the MGD and Mg-stressed plants compared to the control [19]. It is well known that photosynthetic pigment content reduction is always accompanied by photosynthetic inhibition [32], and, while the molecular mechanism needs further exploration, we showed in our earlier report that the chlorophyll content of mulberry seedlings (*M. alba*; Yu-711) under MGD and imbalances (20 d) significantly decreased, while the photosynthetic pigment contents gradually decreased from the interior regions to the edge [19]. Mg remains an essential element as it forms the core center of the Chl molecule in the porphyrin ring and plays a role in activating or regulating various kinases, including ATPases and ribulose-1,5-bisphosphate carboxylase/oxygenase (RuBisCO) [33,34]. As a result, MGD or imbalances can disrupt many physiological processes, including Chl biosynthesis and plant growth and development [34].

An examination of the chloroplast ultrastructure in *M. alba* leaves using transmission electron microscopy (TEM) demonstrated that sufficient Mg concentration was necessary to preserve the chloroplasts’ integrity. Conversely, the chloroplast ultrastructure was compromised to different extents by excessive or inadequate Mg supply. This damage manifested in the rise of starch granules, curved and unsecured stacks, and a decrease in the quantity of substrates (Figure 2). The disruption of the chloroplast was further revealed by the transcriptome analysis. Several light harvesting complex, chlorophyll biosynthesis, and ATP synthase-related genes (*CHLM*, *CAB genes*, *CAP10A*, *PETE*, *HEMA1*, *DVR*, *LHCA6*, *LHCA5*, *LHCB5*, etc.) were altered and downregulated, while a few (*PAO*, *CHLASE1*, *SGR*) were upregulated in response to Mg imbalances (Figure 6). Magnesium protoporphyrin IX methyltransferase (*CHLM*) is an enzyme involved in the biosynthesis of chlorophyll in plants. This gene catalyzes the transfer of a methyl group to magnesium protoporphyrin IX, a key step in the conversion of protoporphyrin IX to chlorophyll. This enzyme plays a crucial role in the regulation of chlorophyll production, which is essential for photosynthesis and plant growth. In our study, the expression of *CHLM* only occurred in MgD (T1) and low Mg supply (T2) (Figure 6), suggesting that MGD or very low supply affected the expression of the key enzymatic gene [6]. Dysfunction of *CHLM* can lead to impaired chlorophyll synthesis and affect the overall development and growth of plants [35]. This led to the chlorosis phenomenon observed in the leaves’ morphology in our earlier report [19] and the damaged chloroplast ultrastructure observed in this study (Figure 2). 

**Figure 13:**
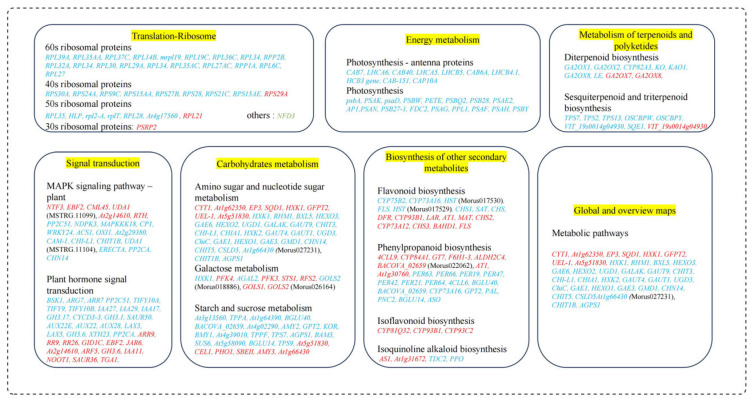
Various mechanisms in *M. alba plants in* response to Mg imbalances. Genes in red and blue fonts are up- and downregulated. Various significant pathways are highlighted in yellow.

Photosynthetic pigment content reduction in response to MGD is a commonly known phenomenon [36]. This situation suggests that prolonged exposure to MGD leads to a decrease in reaction centers linked to the light harvesting complex and photosystem, thus causing a decline in the overall photosynthetic rate and chlorophyll content [36]. In this study, chlorophyll *a*–*b* binding proteins and photosystem I and II reaction center and core complex genes were significantly downregulated in the MGD, low, and toxicity groups (Figure 6) and were significantly involved in energy metabolism pathway (Figure 13). Earlier reports revealed that MGD altered the downregulation of chlorophyll *a–b* binding protein, *CAB2* [10]. Our findings indicate that MGD, low supply, or toxicity affected the mulberry plant development by causing breakdown in photosynthetic pigment and chlorophyll biosynthesis and, hence, activating the downregulation of *CAB40*, *CAB7*, *CAB6A*, *CAB-151*, and *CAP10A* genes and other photosystem I and II reaction center and core complex genes including *LHCA5*, *LHCA6*, and *LHCB5* (Figure 6). It is important for the proper removal of Chl molecules from the chloroplast during the Chl degradation pathway to protect the photosynthetic apparatus from severe growth conditions to ensure plant growth and development [37,38]. This is mediated by an important enzymatic gene including *STAY-GREEN* (*SGR*), pheophorbide *a* oxygenase (*PAO*), chlorophyllase-1 (*CHLASE1*). In this study, the expression levels of *SGR*, *PAO,* and *CHLASE1* were upregulated in the MGD (CK-v-T1), low (CK-v-T2), or toxicity (CK-v-T4) groups (Figure 6). *STAY-GREEN* (*SGR*) is a Mg-dechelatase which is important in the Chl degradation pathway by removing Mg nutrient from Chl degradation to fast-growing vegetative tissues and reproductive organs, hence, ensuring nutrient remobilization [38]. Studies have reported that the absence of *SGR* affected the degradation of *LHCII* subunits [31]; thus, the upregulation of *SGR* in our study could suggest it has a possible role in the Chl degradation process in mulberry during Mg stress, although experimental evidence is needed to ascertain the involvement of the *SGR* gene in Chl degradation in mulberry. In a recent study, it was reported that Mg stress triggers *SGR*-mediated Chl degradation for Mg remobilization [11], which could explain the upregulated expression status of *SGR* in our study. Whereas chlorophyllases are responsible for the removal of Phytol, Pheophorbide a oxygenase (*PAO*) is also important as it is responsible for the oxygenolytic opening of the porphyrin ring of pheide *a* to yield red Chl catabolites (RCC) in the Chl degradation pathway [31]. Expression of *SGR* is said to have influence on *PAO* regulation by modulating *PAO* activity [38], suggesting that the upregulation of these genes (*SGR*, *PAO,* and *CHLASE1*) in our investigation in response to Mg imbalances could prove their critical role in Chl degradation, and this needs to be proved experimentally. Our observation of both up- and downregulation of genes involved in Chl biosynthesis supports the hypothesis that Mg imbalance in *M. alba* altered the genes implicated in the photosynthetic pathway.

### 4.2. Mulberry Response to Mg Imbalances Altered Transcriptional Factors and Signaling Genes

We have revealed in our previous reports that Mg imbalances affected mulberry plants’ physiological and metabolites status by decreasing some prominent morphological and physiological parameters, including biomass, photosynthetic rate, chlorophyll contents, and others [19]. One could speculate that Mg imbalances could perturb the transcriptional and signaling genes when *M. alba* plants are exposed to different levels of Mg. Transcription factors not only integrate both internal and external signals to regulate the transcriptional expression of diverse genes to improve plant stress tolerance but are also known to strictly control plant growth, development, and nutrient acquisition [39,40]. Our transcriptome analysis reveals that transcription factors from *ERF* (*ERF13*, *ERF113*, and *ERF110*), *GT-2*, *NAC047*, *WRKY* (*WRKY69* and *WRKY72A*), *MYB* (*MYB2* and *MYB62*), and *BHLH* (*BHLH130*) families were significantly upregulated in the leaves of induced-Mg-deficiency *M. alba* plants (Figure 10), suggesting that Mg signaling transduction was triggered to compensate for the ripple effects of Mg deficiency. These upregulations in leaves could also imply that members of these TFs families may be implicated in Mg deficiency adaptation in *M. alba*. Similar responses were reported in *N. cadamba* and *Citrus sinensis* under Mg deficiency (MGD) stress, where TF families such as *MYC* (v-myc avian myelocytomatosis viral oncogene homolog), *MYB* (v-myb avian myeloblastosis viral oncogene homolog), *bHLH* (basic helix-loop-helix), and *WRKY* families were markedly upregulated in leaves of MgD-stressed plants [39,40], which is in accordance with our current findings. Intriguingly, the Mg toxicity stress on the other hand triggered a decrease in mRNA levels of TFs, resulting in downregulation of these genes in *M. alba* leaves. For example, TF genes, viz., *ERF053*, *BHLH62*, *BHLH123*, *ERF4*, *MYB25,* and *OFP1,* were downregulated and exclusively expressed in CK-vs-T5, CK-vs-T3, and CK-vs-T4 (Figure 10A,B), highlighting that *M. alba* plants exposed to extreme Mg employ downregulation mechanisms of these TF genes to cope with stress. 

The transcripts’ expression of ethylene-related genes, by coordinating with cascades of environmental cues or signals, can spontaneously modulate growth to counteract the effects of stresses and assist in plant adaptation to variable environments [41]. It has been previously reported that 1-aminocyclopropane-1-carboxylate synthase *(ACS)* enzymes involved in the ethylene biosynthetic pathway (*ASC2*, *ASC7*, *ASC8*, and *ASC11*) observed elevated mRNA levels [42]. Additionally, Liu et al. (2017) also reported two other ethylene synthesis-responsive enzymes, viz., 1-aminocyclopropane-1-carboxylic acid *(ACC)* oxidase and ACC synthase *(ACS)*, with increased levels in roots [43]. Similarly, our results identified *M. alba* genes *Morus012919* and *Morus024218* encoding *ACS1* (1-aminocyclopropane-1-carboxylate synthase 1; XP_010092578.1) and *ACS1* (1-aminocyclopropane-1-carboxylate synthase; XP_010089045.1), respectively (Figure 11B). These two genes underwent different regulation patterns with respect to Mg deficiency and toxicity. The *Morus012919* gene encoding *ACS1* (1-aminocyclopropane-1-carboxylate synthase 1; XP_010092578.1) was upregulated in response to lack of Mg, whereas the *Morus024218 ACS1* (1-aminocyclopropane-1-carboxylate synthase; XP_010089045.1) gene was solely expressed in toxicity but was downregulated (Figure 11). This phenomenon indicates that *M. alba* plants adopt two different mechanisms to adapt and cope with both Mg deficiency and toxicity stresses. Mg stress also triggered the downregulation of genes associated with auxin biosynthesis and transport (*SAUR50*, *AUX22E*, *AUX22*, *AUX28*, *LAX3*, *LAX5*) and signal transduction (*MAPKKK18*, *CP1*, *WRKY24*, *ACSI*, *OXI*, *At2g29380*, *CAM-1*, *CHI-L1*, *CHITIB*, *UDAI* (MSTRG.11104), *ERECTA*, *PP2CA*, *CHN14*) in this study (Figure 13). Earlier reports by Ishfaq et al. (2021) had observed similar patterns in genes related to auxin biosynthesis (*TAR/YUC*), transport (*LAXs*, *PINs*), and signal transduction (*IAAs*, *ARFs*) in magnesium-deficient tomato roots [44]. Furthermore, ABA receptor genes (*PYL1*, *PYL2*, *PYL4*) were mainly found to be downregulated in leaves of *M. alba* plants exposed to lack of Mg in our study (Figure 11A). The expression of these phytohormone- and signal-related genes indicates that *M. alba* downregulates cascades of unique plant hormones and signaling proteins to regulate its growth and tolerance mechanisms of leaves of Mg-deficient plants, which is at odd with those previously chronicled [39].

### 4.3. Mulberry Response to Mg Imbalances Trigger Antioxidants and Cell Wall Defense Genes

The enzyme catalase (CAT) is of utmost importance in safeguarding cells against oxidative harm, as it facilitates the breakdown of hydrogen peroxide into water and oxygen [45]. The present study found that CAT activity increased in the Mg-treated samples, whereas Mg deficiency significantly inhibited the activity of CAT compared to the CK group, even though low-Mg-treated samples increased CAT activity but at a decreasing rate (Figure 1E). Our results agree with [18] who opine that CAT activity decreased in Mg-deficient treated mulberry plants. An increase in CAT activity under low and excessive Mg supply has been reported [46], which agrees with our current results. However, our transcriptome analysis identified the *CAT1* (Catalase isozyme 1; EXC51646.1) gene to be upregulated, with a low expression level exclusively under Mg deficiency (Figure 11A), suggesting possible safeguarding of plant cells against oxidative stress. The elevation of antioxidant enzymes in response to excessive Mg levels can be attributed to the physiological imbalances caused by the surplus of this nutrient, the hindrance in the assimilation of other nutrients, particularly potassium (K), and the escalation in the salinity of the solution [46]. Peroxidase (POD) is an essential antioxidant whose activity level can be used as a biomarker to determine plant membrane integrity. Increasing POD enzyme activity is important in corroborating the induction of oxidative stress [47]. In this study, our results reveal that POD activity decreased in Mg deficiency, low, or excess Mg, but the activity significantly increased in the Mg-sufficiency treated plants (CK) (Figure 1F). This was evident in the MDA content (Figure 1C), indicating that Mg deficiency, low, or excessive supply inhibited POD activity, leading to membrane damage because of ROS production. Our results are also in accordance with what was earlier reported in the mulberry plant under Mg deficiency [18]. To support the premise that sub-optimal and excess Mg reduce the activity of POD in *M. alba*, our transcriptome analysis reveals downregulation of peroxidase genes such as *PER42*, *PER21*, and *PER47* in the low-Mg-treated (CK-vs-T3) and toxicity (CK-vs-T4) groups (Figure 8A,B), signifying alterations and inhibition of POD activity, which resulted in the disruption of membrane integrity, and this situation might have also affected the physiological state of the mitochondria structure as observed in Figure 3. This is because mitochondria are a major source of ROS production, particularly during oxidative phosphorylation. Excessive ROS can lead to oxidative damage to mitochondrial components, including proteins, lipids, and DNA, ultimately affecting mitochondrial structure and function. Peroxidases can help maintain the structural integrity of mitochondria and preserve their function in energy production and other cellular processes, thereby giving the plant a defensive ability. By catalyzing the reduction of superoxide (O^2−^) to hydrogen peroxide (H_2_O_2_), superoxide (SOD) dismutase acts as the initial enzyme in the ROS scavenging pathway [48]. From our results, it was evident that SOD activity was significantly elevated in all the Mg-treated plants irrespective of Mg concentration. Conversely, SOD activity was significantly reduced in the Mg-deficiency plants (Figure 1G). SOD activity being responsive to Mg supply suggests that Mg supply induces an antioxidant metabolism which was active to mitigate the negative effects of high ROS production [45]. Tewari et al. (2006) found that SOD content was increased in Mg-deficient mulberry plants [18]; however, our current study reveals that SOD activity elevated in only Mg-treated mulberry plants. Our study agrees with the report by another study, which stated that an increase in Mg content increased SOD activity compared to Mg-deficient samples [45]. A recent report reveals that high-Mg-treated samples, coupled with steroids, maximized SOD activity in soybean plants, which is concomitant with our results [33].

The mechanism of anthocyanin biosynthesis involves a series of structural molecules and regulatory genes. Dihydroflavonol 4-reductase (*DFR*) is crucial in the mechanistic formation and biosynthesis of anthocyanins and proanthocyanidins in plants [49]. Functionally, *DFR* is known to catalyze the transformation of dihydroflavonols, as well as dihydrokaempferol (*DHK*), dihydroquercetin (*DHQ*), and dihydromyricetin (*DHM*), into leucoanthocyanidins [49]. Dihydroflavonols are substrates or components of the flavonol biosynthesis pathway and are catalyzed by flavonol synthase (*FLS*) [49]. The *DFR* has been a subject of immense interest in recent times due to its substrate-specific nature and difunctional catalytic roles in the anthocyanin biosynthetic pathway in plants. This difunctional catalytic role means that it has two sites in its molecular structure that are highly reactive during catalysis. An overexpression of the *CsDFR* gene in tobacco reveals that transgenic tobacco and transgenic lines demonstrated resistance against drought stress and oxidative stress by scavenging the activities of free radicals [50], suggesting that *DFR* plays a key role in ROS scavenging. A subcellular localization via tobacco infiltration assay indicated that *CnDFR* has a dual localization in the nucleus and cell membrane [50]. In this study, our subcellular localization analysis shows that the *MaDFR* gene is predominantly localized in the cytoplasm of leaves of *M. alba* when exposed to different Mg levels (Figure 12). This indicates that the localization of the *DFR* gene is species-specific, and its localization differed from species to species; however, its role in scavenging ROS could remain same across species. In *Brassica napus*, a total of 26 *BnDFR* genes were localized in chloroplasts, cytoplasm, nuclei, and mitochondria [51], which is in accordance with our study. Interestingly, in our transcriptome analysis, genes (such as *DFR*, *LAR*, *AT1*, *MAT*, *CHS2*, *CYP73A12*, *CHS3*, *BAHD1*, and *FLS)* in the biosynthesis of secondary metabolite pathways were identified to be upregulated after 20 d exposure to different Mg levels (Figure 13), suggesting spontaneous activation of precursors involved in the biosynthesis of various antioxidants and flavonoids, which assist in counteracting the detrimental effects of Mg stresses.

Flavonoids are synthesized from the phenylpropanoid pathways that employ several cytochrome (CYP450) families, such as the *CYP93* gene family, with antioxidation roles in plants stress [52]. Due to its involvement in NADPH- and O_2_-dependent hydroxylation reactions, cytochrome (CYPs) remains the largest enzyme class across plant species [53]. Cytochrome is not only involved in the detoxification of xenobiotics but is also associated with the catalysis of biosynthesis of secondary metabolites, antioxidants, and phytohormones in higher plants [53,54]. In *C. sinensis* leaves, cytochrome P450 (CYP450) was upregulated under Mg deficiency, as reported by Yang et al. (2019) [40]. In this study, several cytochrome family genes were expressed and upregulated in response to varied levels of Mg application. For instance, *CYP71B34* and *CYP71D10* genes were upregulated in Mg deficiency in our study (Figure 7A), indicating their probable roles in antioxidation under Mg deficiency. However, these cytochrome genes, viz., *CYP81Q32*, *CYP84A1*, *CYP78A7*, *CYP81Q32*, *CYP76B6*, and *At4g17280,* were exclusively expressed and upregulated in excess Mg application in this study (Figure 7A,B). The transcriptional expression of a higher number of cytochrome genes under Mg toxicity indicates that *M. alba* plants under Mg stress employed the accumulation of higher mRNA to enhance the mitigation and detoxification of excess Mg. The result of this study is in line with what was reported [52], where *HaCYP93A1* was significantly upregulated under salinity treatment at all three time points in sunflowers. 

We reported in our previous studies that Mg deficiency and toxicity pose direct consequences to plant morpho-physiological parameters, including biomass production, carbon dioxide (CO_2_) fixation, and protection against photooxidative stress, which lead to reduction in yield, growth, and development of plants [19]. For plants to cope with these stresses, plants have evolved cascades of regulatory mechanisms, including the development of unique gene families, viz., Xyloglucan endotransglucosylase/hydrolases (*XTH*s), endoglucanases, pectinesterase, pectin/pectate lyase-like (*PLL*), and polygalacturonase, which are implicated in regulating cell wall plasticity and wall-loosening [55,56]. In this study, cell wall-related genes were altered when plants were exposed to varied Mg levels for a period of 20 d. For example, with the exception of induced-Mg-deficiency plants, cell wall genes such as *EP3* (endochitinase EP3; XP_010094102.1), *GATL4* (probable galacturonosyltransferase-like 4; XP_010105689.1), and *WAT1* (plant-drug/metabolite exporter; MSTRG.6737) were significantly upregulated in the other treatment, including toxicity (Figure 8A), highlighting that these genes might be involved in the response of growing cells to cellulose and maintenance of cell wall integrity. Nishikubo et al. (2011) reported that transgenic hybrid aspen overexpressing *PtxtXTH34* had increased vessel diameter [57]. Similar responses were also recorded [58] in Arabidopsis, where *AtXTH4* and *AtXTH9* significantly regulated wood cell expansion and secondary wall formation. Additionally, upregulation of 7 *PMEs*, 11 *XTHs*, 4 *TLPs*, 6 *LACs*, 10 expansins, 6 *GLPs*, 8 *XCPs*, 9 subtilisin-like proteases, 9 endoglucanases, and 1 *SKU5* were observed in induced-Mg-deficiency *Citrus sinensis* plants [56]. However, xyloglucan endotransglucosylase/hydrolase (*XTH33*, *XTH16*, *XTH6*), and pectinesterase/pectinesterase inhibitor (*PME44*, *PME40*, *PME41*, *PME18*, *PME51*, *PME43*, *PME54*, etc.) genes isoforms were predominantly downregulated in the moderate Mg treated (CK-vs-T3) and toxicity (CK-vs-T4) groups (Figure 8A,B), and might be responsible for the decrease in lignification, wood cell expansion and secondary wall formation. Our findings are at odds with what was reported by Xin et al. (2021) in induced-Mg-deficiency *C. sinensis* plants [56]. The alteration of several antioxidant and cell wall, TFs, and signaling genes in this study corroborates with our earlier established hypothesis that Mg imbalances perturb the metabolic pathways of defense mechanisms in *M. alba* plants to cope with the Mg stresses.

## 5. Conclusions

In conclusion, any Mg level (imbalance), apart from optimal, in part leads to disruption of the photosynthetic apparatus, including disorientation and disorganization of the chloroplast and mitochondria ultrastructural organs, leading to a drastic reduction in growth and yield of *M. alba* plants. This dysfunctional growth altered by Mg imbalances triggered numerous genes and mechanistic processes and pathways including upregulation and downregulation of genes involved in chlorophyll biosynthesis, protein modification-ubiquitin, cytochrome, carbohydrate, and cell wall defense and signaling in the *M. alba* plant. This study shed light and provided insights into the *M. alba* transcriptome in response to Mg imbalance and can assist in the breeding of Mg deficiency- and toxicity-tolerant and magnesium-use-efficiency (MUE) genotypes and cultivars. This study is limited by its use of only one mulberry cultivar and late Mg imbalances samples for gene detection, making it limited in scope.

## Figures and Tables

**Table 1:** List of some DEGs linked to a specific group of Mg supply or Mg-deficiency treatments.

**DEGs Response to Mg Deficiency (T1)**				
**Gene ID**	**Symbol**	**log2 FC**	**Regulation**	**Description**
Morus011995	*PME6*	−1.14	Down	pectinesterase
Morus013583	*CML46*	−2.82	Down	putative calcium-binding protein
Morus010081	*NPF2.11*	1.01	Up	protein NRT1/PTR FAMILY 2.11
Morus027772	*BCA2*	−1.27	Down	carbonic anhydrase 2 isoform X1
Morus015014	*BCA2*	16.44	Up	carbonic anhydrase 2
Morus015626	*CODM*	4.52	Up	codeine O-demethylase
Morus021157	*ValCS*	1.59	Up	valencene synthase
Morus008503	*ACR2*	6.25	Up	ACT domain-containing protein
Morus003982	*SAR1A*	−10.16	Down	GTP-binding protein
Morus012609	*F3H-3*	10.13	Up	Leucoanthocyanidin dioxygenase
**DEGs Response to Low Mg Supply (T2)**				
**Gene ID**	**Symbol**	**log2FC**	**Regulation**	**Description**
Morus026743	*SAMT*	1.73	Up	salicylate carboxymethyltransferase
Morus020016	*CAB-151*	−1.41	Down	chlorophyll a-b binding protein 151
Morus018739	*LHCB3*	−1.13	Down	chlorophyll a-b binding protein 3
Morus015887	*PSB28*	−1.38	Down	photosystem II reaction center
Morus004891	*PHOS34*	−1.05	Down	universal stress protein
Morus015920	*PSAE2*	−1.14	Down	photosystem I reaction center subunit IV
Morus003430	*PUB35*	1.36	Up	U-box domain-containing protein 35
Morus025032	*ISA2*	1.67	Up	isoamylase 2
Morus023478	*MYB5*	2.82	Up	myb-related protein 308
Morus016608	*INPS1*	1.11	Up	inositol-3-phosphate synthase
**DEGs Response to Low Mg Supply (T3)**				
**Gene ID**	**Symbol**	**log2FC**	**Regulation**	**Description**
Morus006609	*At2g02240*	10.17	Up	F-box protein
Morus024260	*CYP86A22*	1.1	Up	cytochrome P450 86A22
Morus012299	*NPF5.1*	1.59	Up	protein NRT1/PTR FAMILY 5.1
Morus015082	*DRMH1*	1.18	Up	auxin-repressed 12.5 kDa protein
Morus019005	*NDUFAF6*	10.23	Up	UPF0551 protein C8orf38
Morus017222	*ANN5*	10.31	Up	Annexin D5
Morus017242	*PRR73*	−1.85	Down	Two-component response regulator-like protein
Morus007083	*CSLC1*	−2.59	Down	probable xyloglucan glycosyltransferase 12
Morus000280	*MPE3*	−1.42	Down	Pectinesterase 3
Morus010246	*CYCA3-1*	−9.5	Down	Putative cyclin-A3-1
Morus007976	*JMJ30*	−1.6	Down	lysine-specific demethylase
**DEGs Response to High Mg Supply (T4)**				
**Gene ID**	**Symbol**	**log2FC**	**Regulation**	**Description**
MSTRG.4564	*EX1*	−11.86	Down	protein EXECUTER 1
Morus012479	*PLPBP*	−1.02	Down	pyridoxal phosphate homeostasis protein
Morus002464	*SCPL31*	−9.38	Down	serine carboxypeptidase-like 31
Morus004986	*At1g73050*	−9.38	Down	(R)-mandelonitrile lyase-like
Morus010123	*SPL13A*	−2.2	Down	squamosa promoter-binding-like protein 13A
Morus009607	*SPL13*	3.82	Up	squamosa promoter-binding protein 1
Morus021834	*HAT3*	1.16	Up	homeobox-leucine zipper protein
Morus027660	*CYP71B10*	1.99	Up	cytochrome P450 71B10
Morus017881	*RBP47B*	1.01	Up	Polyadenylate-binding protein
Morus025208	*DOF3*	1.06	Up	dof zinc finger protein
**DEGs Response to High Mg Supply (T5)**				
**Gene ID**	**Symbol**	**log2FC**	**Regulation**	**Description**
Morus025314	*SMO2-2*	−1.01	Down	Methylsterol monooxygenase 2-2
Morus024401	*LOG2*	−1.06	Down	E3 ubiquitin-protein ligase MGRN1
Morus002502	*MYB3R4*	−3.45	Down	transcription factor MYB3R-1
Morus006501	*CYP735A2*	−9.14	Down	Cytochrome P450 734A1
Morus011257	*CYP73A16*	−1.56	Down	trans-cinnamate 4-monooxygenase
Morus008671	*NPF5.2*	1.22	Up	Peptide transporter
Morus003782	*WRKY50*	2.34	Up	probable WRKY transcription factor 50
Morus000989	*CYP78A7*	4.24	Up	cytochrome P450 78A7
Morus002784	*WRKY48*	1.37	Up	probable WRKY transcription factor 48
Morus021252	*WAKL14*	1.72	Up	Wall-associated receptor kinase-like 14

## Data Availability

The data used are described in this study. Raw RNA-Seq data have been deposited at NCBI SRA database with the link: (https://ncbi.nlm.nih.gov/subs/sra; accession number: PRJNA951543; accessible on 31 December 2024). Further inquiries can be made to the corresponding author (s) on reasonable request.

## Supplementary Materials

The following supporting information can be downloaded at: https://www.mdpi.com/article/10.3390/antiox13050516/s1, Excel file: Tables S1–S8. Word file: Figures S1–S5.
