# Supplementary material for: Comparative Physio-Biochemical and Transcriptome Analyses Reveal Contrasting Responses to Magnesium Imbalances in Leaves of Mulberry (Morus alba L.) Plants
Source: Antioxidants (Basel). 2024 Apr 25;13(5):516. doi: 10.3390/antiox13050516 (PMC11117640; doi:10.3390/antiox13050516)
Supplement: Supplementary file 1 [file antioxidants-13-00516-s001.zip › Addtional file figures S1-S5.pdf]

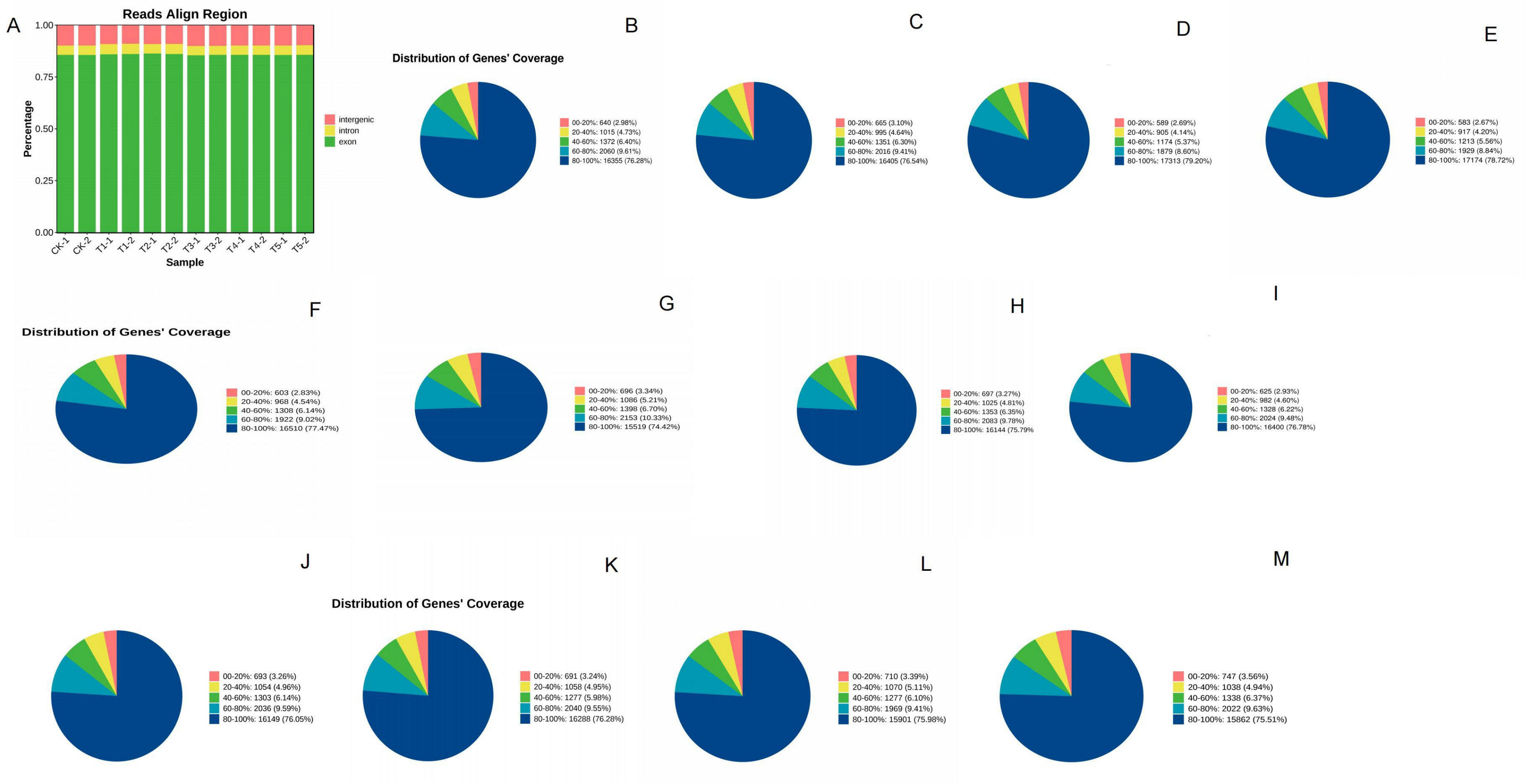

Figure S1. (A) alignment of the sample's clean reads to the reference genome. (B, C) genes coverage distribution in CK-1, CK-2. (D, E) genes coverage distribution in T1-1, T1-2. (F, G) genes coverage distribution in T2-1, T2-2. (H, I) genes coverage distribution in T3-1, T3-2. (J, K) genes coverage distribution in T4-1, T4-2. (L, M) genes coverage distribution in T5-1, T5-2. Mg sufficiency (CK; 3 mM), deficiency (T1; 0 mM), low (T2; 1 mM) moderate low (T3; 2 mM) toxicity (T4; 6 mM) and high toxicity (T5; 9 mM).

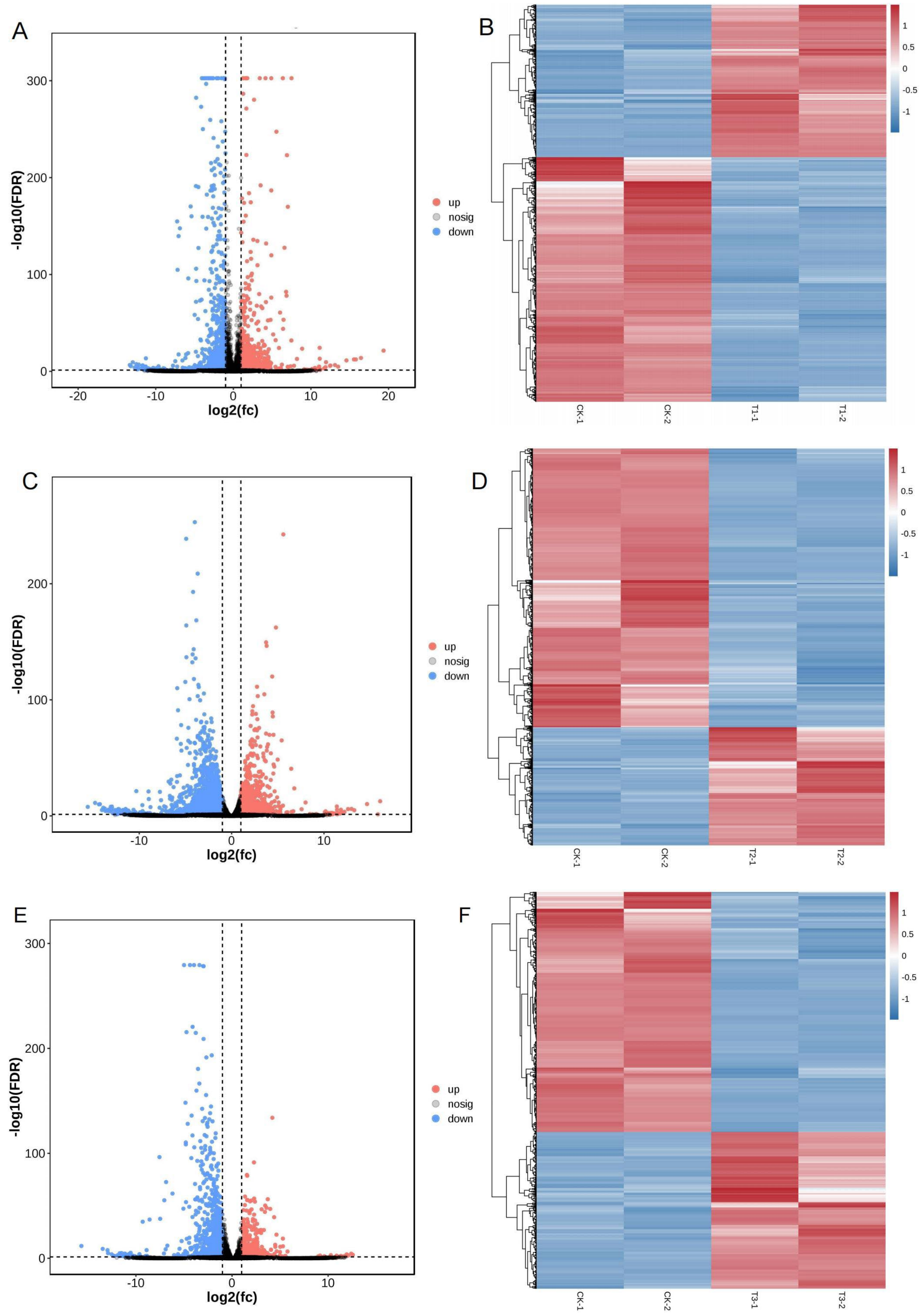
A
-log10(FDR)
log2(fc)
up
nosig
down
B
CK-1
CK-2
T1-1
T1-2
C
-log10(FDR)
log2(fc)
up
nosig
down
D
CK-1
CK-2
T2-1
T2-2
E
-log10(FDR)
log2(fc)
up
nosig
down
F
CK-1
CK-2
T3-1
T3-2

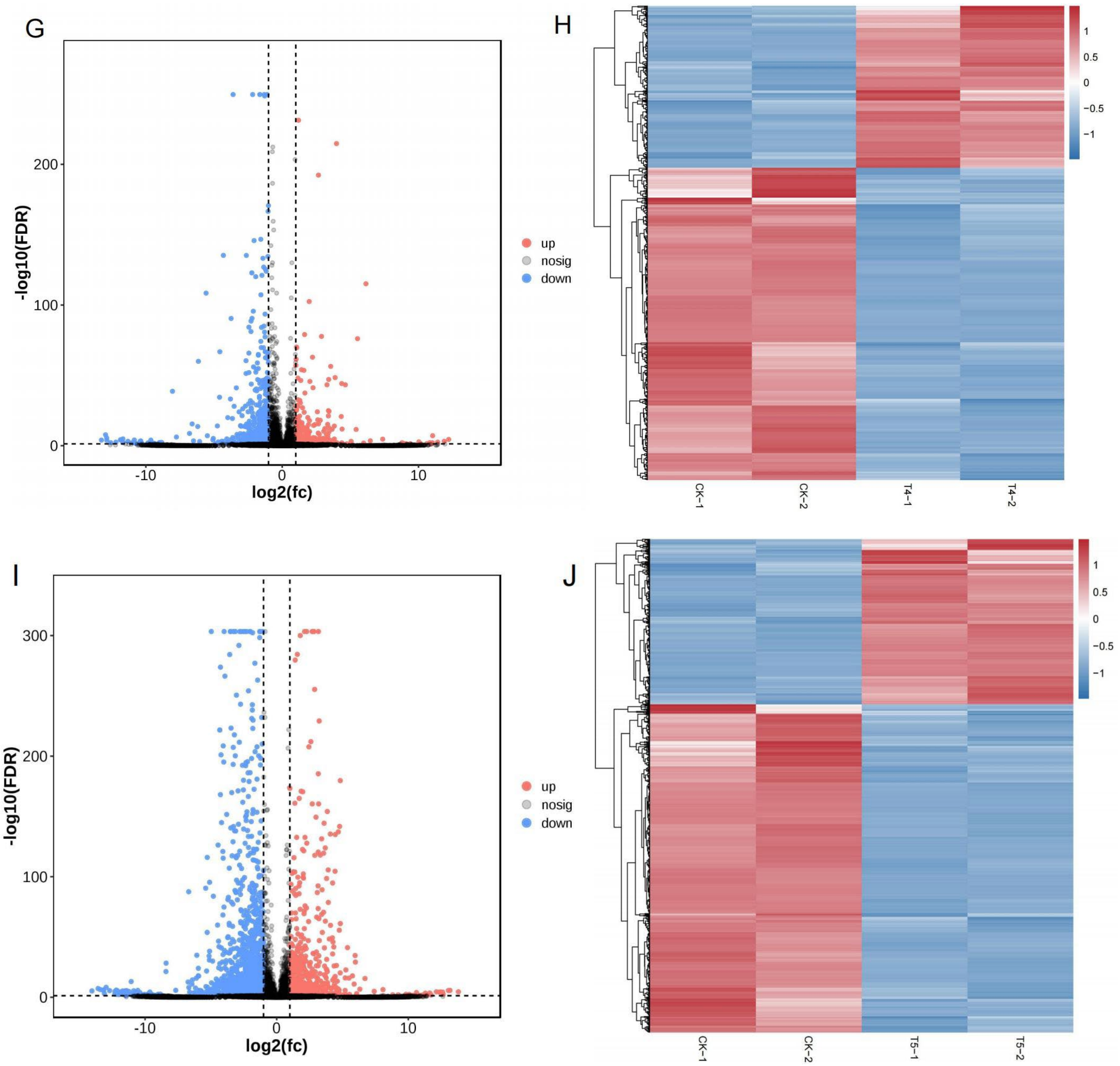

Figure S2. Volcano plot and heatmap showing the distribution of the deferentially expressed genes (DEGs) in T1 (A, B), T2 (C, D), T3 (E, F), T4 (G, H) and T5 (I, J). Red and blue colors represent genes with higher and lower concentrations, respectively.

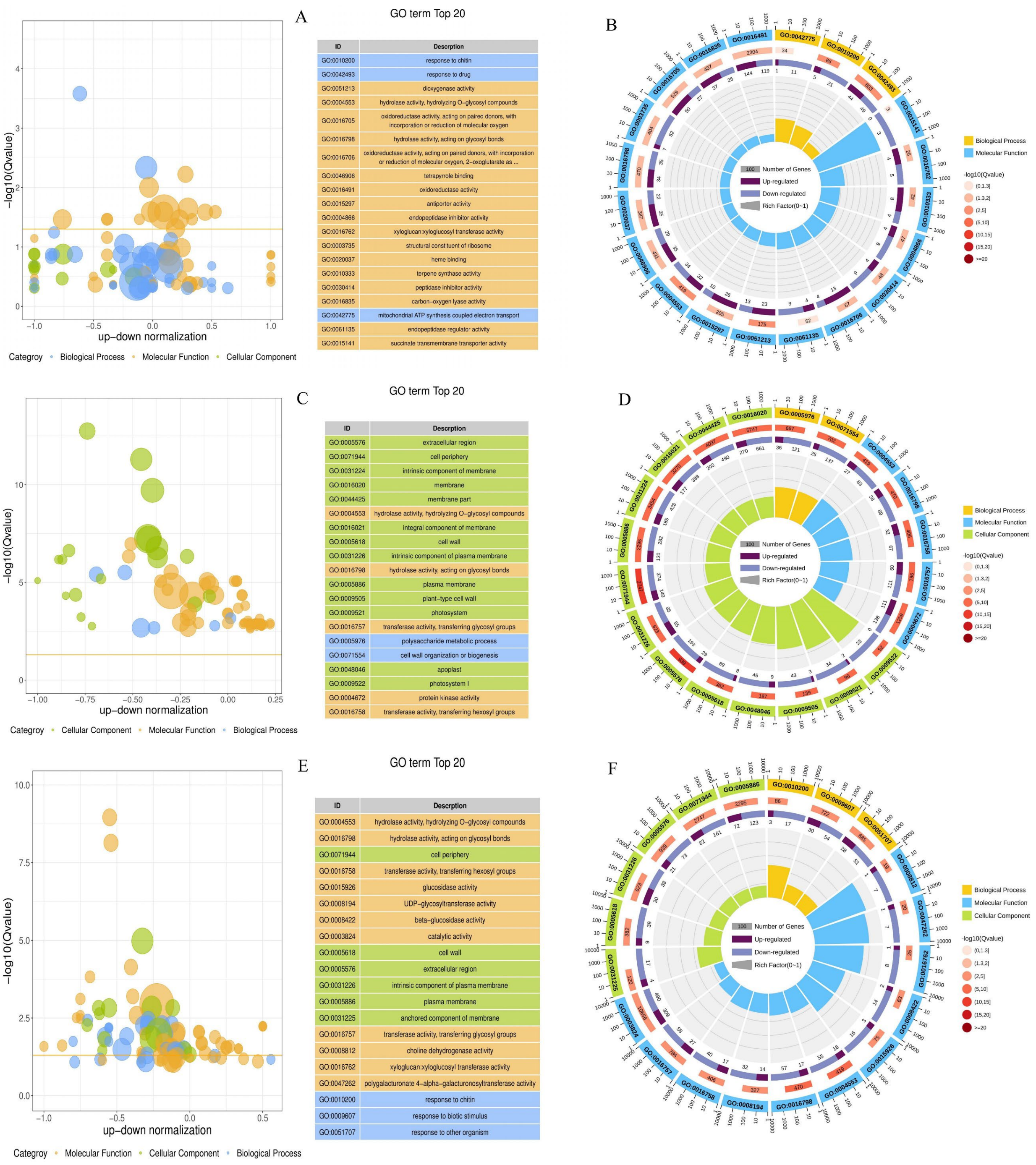

Figure S3. Gene Ontology (GO) enrichment results of the DEGs in deficiency and low Mg treatments based on q-value <0.05. (b) and enrichment factor. (A, B) Top 20 GO enrichment in T1. (C, D) Top 20 GO enrichment in T2. (E, F) Top 20 GO enrichment in T3.

A

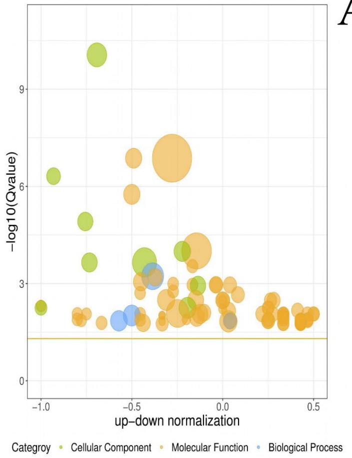

GO term Top 20

ID	Descrption
GO:0005576	extracellular region
GO:0003824	catalytic activity
GO:0004553	hydrolase activity, hydrolyzing O−glycosyl compounds
GO:0048046	apoplast
GO:0016798	hydrolase activity, acting on glycosyl bonds
GO:0005618	cell wall
GO:0016740	transferase activity
GO:0031226	intrinsic component of plasma membrane
GO:0071944	cell periphery
GO:0030312	external encapsulating structure
GO:0030414	peptidase inhibitor activity
GO:0005975	carbohydrate metabolic process
GO:0008194	UDP−glycosyltransferase activity
GO:0016758	transferase activity, transferring hexosyl groups
GO:0004866	endopeptidase inhibitor activity
GO:0015144	carbohydrate transmembrane transporter activity
GO:1901476	carbohydrate transporter activity
GO:0061134	peptidase regulator activity
GO:0051119	sugar transmembrane transporter activity
GO:0005887	integral component of plasma membrane

B

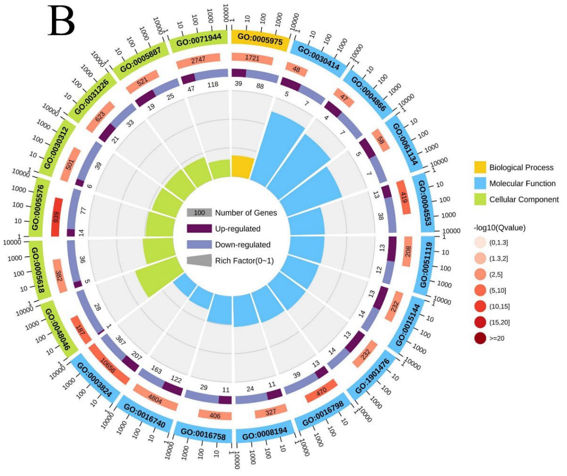

C

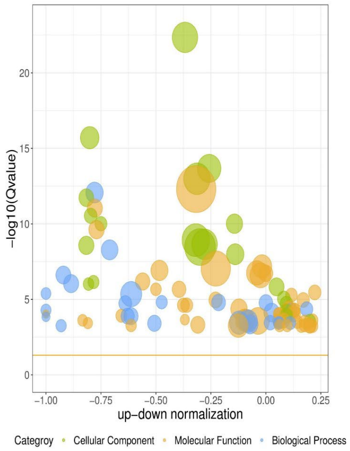

GO term Top 20

ID	Descrption
GO:0071944	cell periphery
GO:0005576	extracellular region
GO:0005886	plasma membrane
GO:0031224	intrinsic component of membrane
GO:0003824	catalytic activity
GO:0071554	cell wall organization or biogenesis
GO:0005618	cell wall
GO:0004553	hydrolase activity, hydrolyzing O−glycosyl compounds
GO:0048046	apoplast
GO:0009505	plant−type cell wall
GO:0031226	intrinsic component of plasma membrane
GO:0016798	hydrolase activity, acting on glycosyl bonds
GO:0044425	membrane part
GO:0016021	integral component of membrane
GO:0030312	external encapsulating structure
GO:0016020	membrane
GO:0005976	polysaccharide metabolic process
GO:0044459	plasma membrane part
GO:0004672	protein kinase activity
GO:0016740	transferase activity

D

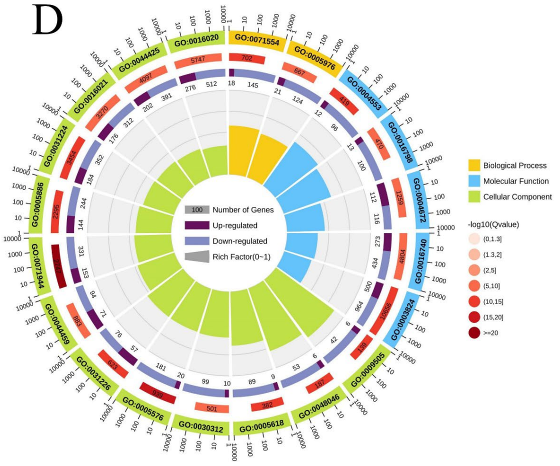

E

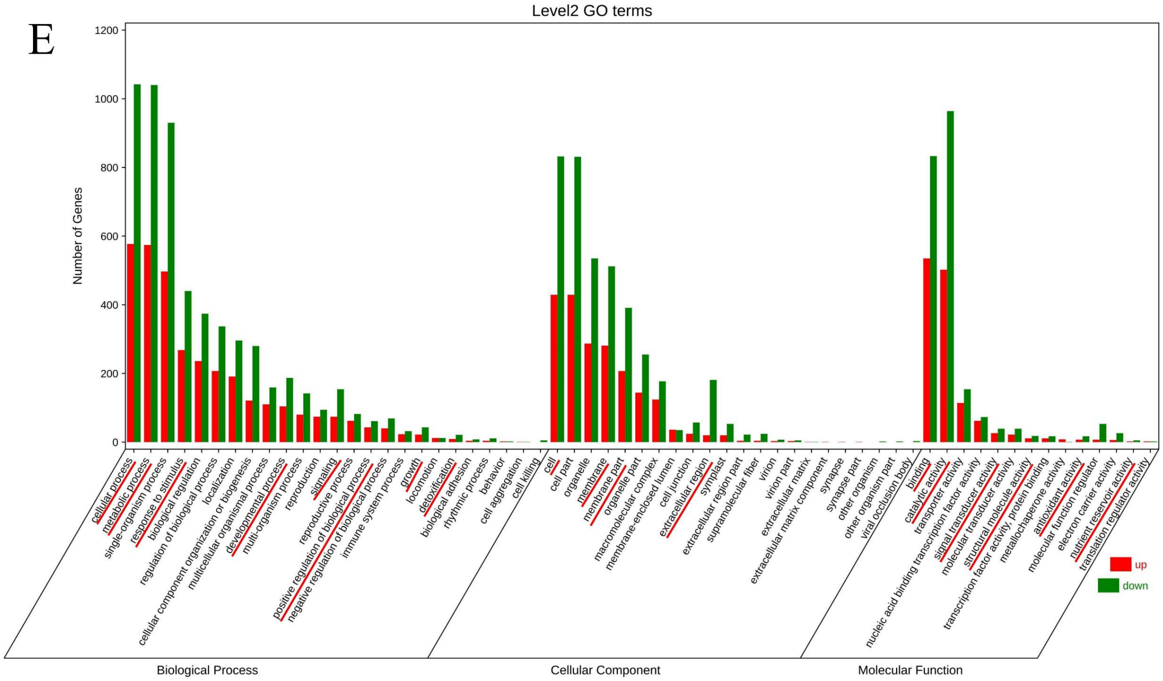

Figure S4. Gene Ontology (GO) enrichment results of the DEGs Mg toxicity treatments based on q-value <0.05. (b) and enrichment factor. (A, B) Top 20 GO enrichment in T4. (C, D) Top 20 GO enrichment in T5. (E) GO term secondary classification involving the DEGs

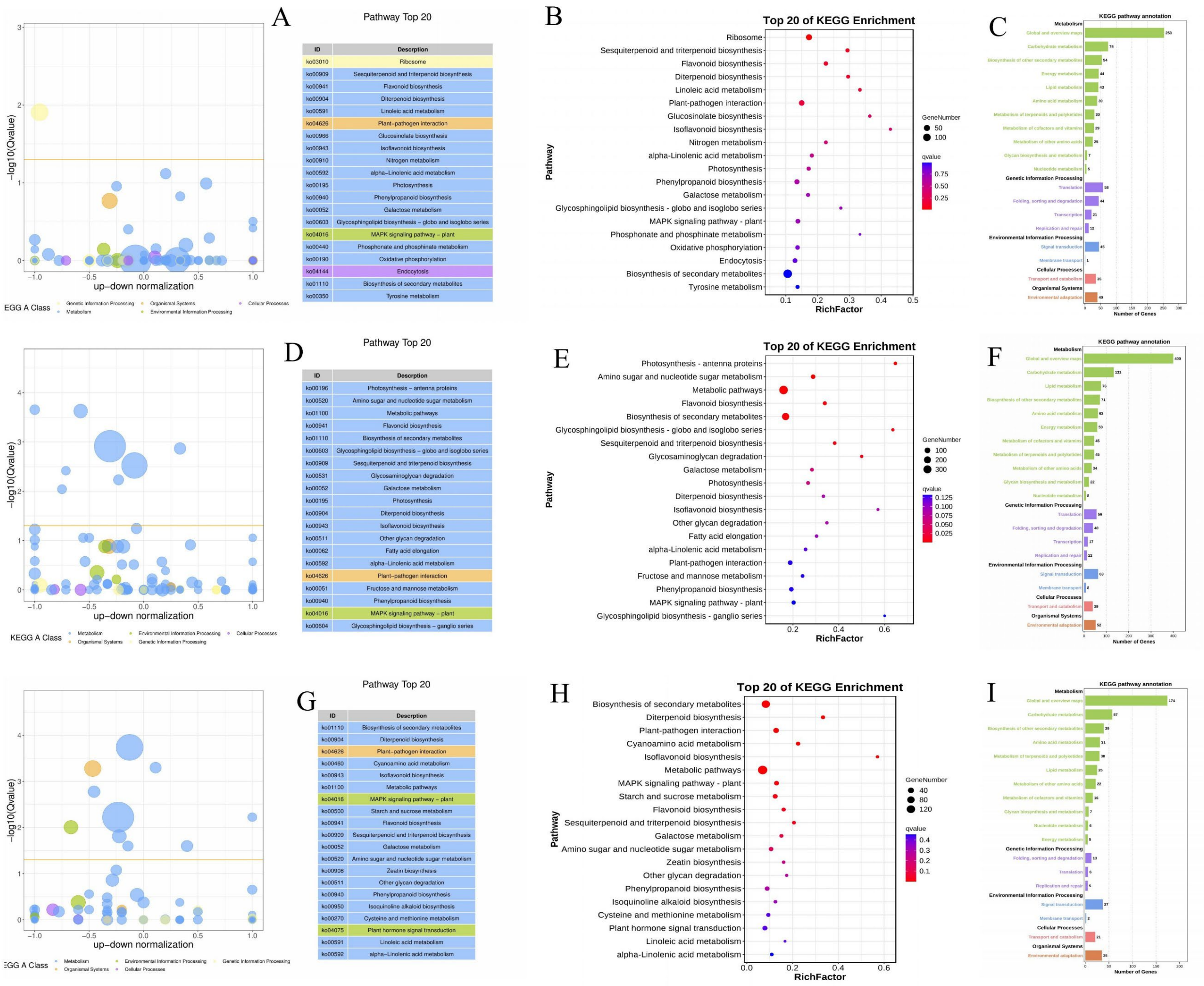

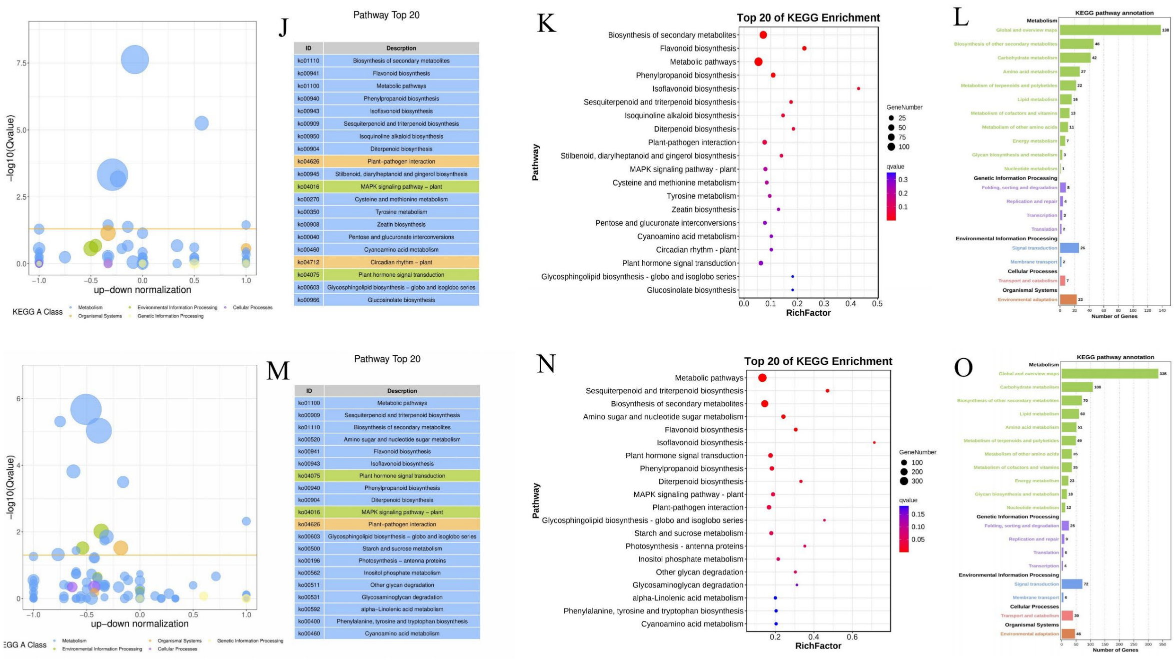

Figure S5. KEGG enrichment analysis of the DEGs across Mg-deficiency and Mg supply treatments. Rich Factor represents the ratio between the number of DEGs mapped to a certain pathway and total number of genes mapped to the corresponding pathway. (A-C) KEGG pathways that are significantly enriched base on q-value (FDR ≤ 0.05) in T1. (D-F) KEGG pathways in T2. (G-I) KEGG pathways in T3. (J-L) KEGG pathways in T4. (M-O) KEGG pathways in T5.
